# Lanatoside C Induces G2/M Cell Cycle Arrest and Suppresses Cancer Cell Growth by Attenuating MAPK, Wnt, JAK-STAT, and PI3K/AKT/mTOR Signaling Pathways

**DOI:** 10.3390/biom9120792

**Published:** 2019-11-27

**Authors:** Dhanasekhar Reddy, Ranjith Kumavath, Preetam Ghosh, Debmalya Barh

**Affiliations:** 1 Department of Genomic Science, School of Biological Sciences, Central University of Kerala, Tejaswini Hills, Periya (P.O) Kasaragod 671316, Kerala, India; dhanasvims@gmail.com; 2 Department of Computer Science, Virginia Commonwealth University, Richmond, VA 23284, USA; preetam.ghosh@gmail.com; 3 Centre for Genomics and Applied Gene Technology, Institute of Integrative Omics and Applied Biotechnology (IIOAB), Nonakuri, Purba Medinipur 721172, West Bengal, India; dr.barh@gmail.com

**Keywords:** Cardiac glycosides, Na^+^/k^+^-ATPase, G2/M phase, apoptosis, autophagy, molecular docking

## Abstract

Cardiac glycosides (CGs) are a diverse family of naturally derived compounds having a steroid and glycone moiety in their structures. CG molecules inhibit the α-subunit of ubiquitous transmembrane protein Na^+^/K^+^-ATPase and are clinically approved for the treatment of cardiovascular diseases. Recently, the CGs were found to exhibit selective cytotoxic effects against cancer cells, raising interest in their use as anti-cancer molecules. In this current study, we explored the underlying mechanism responsible for the anti-cancer activity of Lanatoside C against breast (MCF-7), lung (A549), and liver (HepG2) cancer cell lines. Using Real-time PCR, western blot, and immunofluorescence studies, we observed that (i) Lanatoside C inhibited cell proliferation and induced apoptosis in cell-specific and dose-dependent manner only in cancer cell lines; (ii) Lanatoside C exerts its anti-cancer activity by arresting the G2/M phase of cell cycle by blocking MAPK/Wnt/PAM signaling pathways; (iii) it induces apoptosis by inducing DNA damage and inhibiting PI3K/AKT/mTOR signaling pathways; and finally, (iv) molecular docking analysis shows significant evidence on the binding sites of Lanatoside C with various key signaling proteins ranging from cell survival to cell death. Our studies provide a novel molecular insight of anti-cancer activities of Lanatoside C in human cancer cells.

## 1. Introduction

In Asia, about 8.7 million cases were reported by the end of 2018 with more than 100 different types of cancers, among which 1.57 million reports are from developing countries such as India. Breast and lung cancers are the leading causes for the number of cancer-related deaths [1]. Regrettably, cancers exhibit poor survival rates even after continuous treatment, showing more resistance to conservative cytotoxic agents and ineffectiveness of drugs. Consequently, developing an active therapeutic approach to treat advanced forms of cancer is always an essential issue [2]. Cardiac glycosides (CGs) are among such therapeutic options, which are profusely available from plant and animal sources [3] and clinically used to treat congestive heart diseases. Recent epidemiological studies showed the anti-cancer and anti-viral activities of CGs in several types of cancers and viral diseases [4]. The available CGs are known to act as inhibitors of Na^+^/K^+^ ATPase, which was reported as a prominent drug target for treating cancers [5].

The fundamental mechanism of action of CGs is to bind and inhibit the Na^+^/K^+^ ATPase pump which controls the in and outflow of Na^+^, K^+^_,_ and Ca^+^ during muscle contraction in the heart [6,7]. The chemical core structure of CGs contains a steroid nucleus along with four rings in it. The lactone ring at C17 position controls the pharmacodynamics properties of CGs. Some of the CGs are reported to have anti-proliferative and anti-migratory effects on different types of cancer cells at nanomolar concentrations with possible mechanisms including inhibitions of general protein synthesis and topoisomerase I and II, along with activation of transcription factors like NF-kB [8,9].

It has been reported that minor changes in the core structure of CGs could lead to a loss or gain of function, and it is necessary to evaluate the anti-cancer potential of many such compounds [10]. In this study, we seek to identify the cytotoxicity of Lanatoside C in human cancers along with their mechanism of action and significant role in multiple signaling pathways leading to apoptosis. In addition, it has been reported to inhibit the Na^+^/K^+^-ATPase pump [4]. Recent reports on Lanatoside C have suggested that it possesses anti-cancer activity in glioblastoma, gastric cancer, and colorectal cancer cells [11,12,13]. The anti-viral activity of Lanatoside C was also reported against dengue viral infections [14,15].

Cellular signaling pathways are strongly interconnected with each other to facilitate multicellular functions such as cell proliferation, cell fate determination, and cell migration [16,17]. Inappropriate signal transfer within the cell could lead to pathologies such as cancer, and this dysregulation leads to incorrect signal transmission pathways including PI3K/AKT/mTOR signaling, Wnt signaling, nuclear factor-κB signaling, and many other protein degradation pathways [18,19]. The MAPK and PI3K-mTOR pathways, in combination, act as common mechanisms for maintaining cell survival, proliferation, metabolism, and other cellular functions against extracellular signals [20]; these pathways are known to regulate very highly in comparison to the activity of other pathways.

CGs such as Digoxin, Ouabain, and Digitoxin were clinically approved for their use in cardiac diseases. Nevertheless, several studies have already reported the beneficial properties of these compounds in cancer treatment [21,22]. The present study investigates the anti-cancer activity of Lanatoside C in breast, lung, and liver cancer cells. Specifically, we evaluated the role of Lanatoside C in modulating MAPK, PI3K/AKT/mTOR, JAK-STAT, and Wnt/β-catenin pathways by *in-vitro* and *in-silico* studies.

## 2. Materials and Methods

### 2.1. Cell Lines and Chemicals

Human breast cancer (MCF-7), lung cancer (A549), and hepatocellular carcinoma (HepG2) cell lines were purchased from CSIR-Central Drug Research Institute (Lucknow, India) and normal lung (L132) and liver (WRL68) cell lines were purchased from the National Center for Cancer Cell lines (NCCS, Pune, India). All the cells were cultured in DMEM supplemented with 10% FBS (fetal bovine serum), L-glutamine (2 μM) and antibiotic-antimycotic solution, and incubated at 37 °C in a humidified atmosphere of 5% CO_2_. Lanatoside C was purchased from Sigma-Aldrich (St. Louis, MO, USA) and dissolved in dimethyl sulfoxide (DMSO) by maintaining the overall DMSO concentration not exceeding 0.001% in all the experiments. MTT, Propidium iodide, and TRIzol were purchased from Invitrogen (Carlsbad, CA, USA). In every experiment, the control contained the highest DMSO percentage (0.001%). Peripheral blood mononuclear cells (PBMC) were used for checking the toxicity of Lanatoside C with a wide range of concentrations (0.01–500 µM). PBMCs were purchased from Himedia, Cat#CL003-25 (Mumbai, India). The cells were then revived in the RPMI medium supplemented with 10% FBS and antibiotics. Approximately 1 × 10^5^ cells were seeded in 96 well plates; after 2–4 h incubation, the cells were treated with a wide range of Lanatoside C concentrations to check the toxicity. The experiment was done thrice and results were interpreted in Origin 9.5.

### 2.2. Cytotoxicity Assay

Approximately 3500 cells were seeded in each well of 96 well plates and allowed to attach overnight (16 h). The cells were treated with Lanatoside C with different doses for 24 h. Then, 0.5 mg/mL of MTT solution was added to the cells and allowed to incubate in the dark for 2–4 h, and the dye was dissolved in DMSO. The absorbance was measured at 570 nm and the baseline correction was set to 630 nm.

### 2.3. DNA Damage Assay

DNA damage has been evaluated by comet assay with minor modifications from [23]. Briefly, around 1000 cells were seeded in a 6 well plate and allowed to incubate for at least 16 h. The cells were then treated with inhibitory concentrations for 24 h. After 24 h, cells were harvested and mixed in 0.6 mL of PBS. 1% low melting agarose was prepared and mixed with cells and layered on scored glass slide without forming air bubbles. The slides were then allowed to dry in the air and incubated in lysis buffer overnight. Next, the slides were washed with 1 × TAE three times at 20 min intervals and subjected to electrophoresis at 0.6 V/cm for 25 min. The slides were then stained with 2.5 µg/mL of propidium iodide and washed and distilled for destaining. The cells were visualized for DNA damage using a fluorescent microscope under 20 × magnification (Leica DMI-3000I microscope- Wetzlar, Germany).

### 2.4. Cell Cycle Analysis By Flow Cytometry

DNA content based cell cycle regulation analysis was performed as follows: Briefly, 1 × 10^5^ cells were seeded in a 6 well plate and incubated overnight. After 24 h, the media was removed and the cells were treated with inhibitory concentrations for 24 h. Cells were then trypsinized and centrifuged at 3000g for 5 min and the pellet was dissolved in ice-cold ethanol and stored at −20 °C for a minimum of 24 h. The cells were then washed thrice with PBS to remove ethanol content and incubated at 37 °C with RNase A. The cells were then stained with 0.5 µg/mL of propidium iodide for 30 min and subjected to FACS instrument (BD Biosciences- Allschwil, Switzerland) for cell cycle analysis.

### 2.5. Real-Time PCR Analysis

Total RNA was extracted using TRIzol^®^ (Invitrogen- Carlsbad, CA, USA) reagent by following the manufacturer instructions. A total of 2 µg RNA was used for cDNA synthesis (Verso cDNA synthesis kit, Thermo Fisher Scientific- Waltham, MA, USA) according to the given protocol. Real-time quantitative PCR was performed by using the Origin 2 × SYBR green master mix (Origin, Kerala, India) in Roche light cycler 480 II (Roche) system. RNA expression levels were normalized by using *GAPDH* as the reference gene and calculated using the 2^–∆∆Ct^ method. All the primers used in this study were listed in Table S1.

### 2.6. ELISA (Enzyme-Linked Immunosorbent Assay)

Briefly, the cells were treated with lethal doses of Lanatoside C for 24 h and the total cell lysate was extracted with ROPA lysis buffer (Thermo Fisher Scientific- Waltham, MA, USA). To understand the role of Lanatoside C in MAPK signaling, we performed ELISA with PathScan^®^ MAP Kinase Multi-Target Sandwich ELISA Kit (Cell Signaling Technology, #7274- Danvers, MA, USA) and ELISA antibodies for Caspase 3, 7, and 9 were also purchased from Cell Signaling Technology (Danvers, MA, USA). The experimentation was done by following the manufacturer guidelines. The absorbance was measured at 450 nm. The experiment was repeated three times and the obtained results were plotted in bar charts.

### 2.7. Immunoblotting Studies

After specific treatment incubation, the cells were collected and lysed in RIPA buffer with protease inhibitor cocktail (Roche). The total protein concentration was estimated by using Bradford protein estimation assay and an equal concentration (30 µg) was loaded in each well of 12% SDS-PAGE. After running, the gel was transferred onto the PVDF membrane (Merck Millipore) by using the Trans-Blot^®^ Turbo™ Blotting System (Bio-Rad-Hercules, CA, USA). The membrane was then blocked with 5% Bovine serum albumin (BSA) in TBST for 1 hour at room temperature and then incubated with primary antibody overnight at 4℃. The membrane was washed with TBST 4–5 times and incubated with secondary antibody for 1 h at room temperature. Primary antibodies used in this study were CHK1, CHK2, Cyclin D1, p53, AKT, p38, MEK1, mTOR, p62, PI3K, LC3, CDK6, SAPK/JNK, STAT3, GSK3α, β-catenin, Beclin, and GAPDH (Elabsciences, Houston, TX, USA). The chemiluminescent signals were detected and processed in C-Digit Chemiluminescent Western Blot Scanner (LI-COR Lincoln, NE, USA).

### 2.8. Immunostaining Studies

Approximately 0.3 × 10^6^ cells were seeded on top of the coverslips in six-well plates. After incubation, the cells were treated with lethal doses of Lanatoside C for 24 h. The cells were then fixed with 4% paraformaldehyde and for 0.1% Triton × for 20 min, respectively. After washing the coverslips with 1 × TBS 4–5 times, the coverslip was blocked with 5% BSA for 1 h at room temperature. The coverslip was then incubated with primary antibody overnight at 4 °C. The coverslip was then washed with 1 × TBS and incubated in a secondary antibody (Alexa flour 488- Invitrogen, Carlsbad, CA, USA) for 2 h. Then the coverslip was washed and incubated with 0.1 µg/mL concentration of DAPI for 20 min in the dark and washed 5–6 times with 1 × TBS. The coverslip was then transferred to clean glass slides coated with ProLong Gold Antifade Mountant. An excess amount of antifade was removed and the slides were sealed with wax and observed under a fluorescent microscope with 60 × Magnification (Leica DMI3000- Wetzlar, Germany).

### 2.9. In-silico Docking Analysis

Using built-in-ligand preparation wizard of Discovery studio V3.1. Hydrogen atoms, probable tautomers, low energy ring confirmers, and isomers were produced. Aromaticity has been preserved for both the compounds and pH (6.5–8.5) based ionization was applied. Energy minimization was performed by applying the CHARMm force field for dihedral angles and exact bond length. The 2D-chemical structures of Lanatoside C were downloaded from PubChem (656630) and exported to Discovery Studio V3.1 (San Diego, CA, USA) for the generation of 3D-structure; the structure was optimized using CHARMm force field and was minimized using RMS gradient energy with 0.001 kcal/mol by keeping all the other parameters at default [24]. Crystal structures of STAT3 (PDB:1BG1), PARP (PDB: 1WOK), p38 alpha (PDB: 1OVE), NF-kB (PDB:1VKX), AKT (PDB: 2JDO), CDK6 (PDB: 1X02), mTOR (PDB:4JSP), MKK4 (PDB:3ALN), JAK (PDB:3EYG), BCL-2 (PDB: 2O21), MEK1 (PDB: 3VVH), CHK1 (PDB: 2E9P), PI3K (PDB:3L54), and CHK2 (PDB: 2WTJ) protein structures were retrieved from Protein Data Bank (PDB) and imported to Discovery studio work environment. The structure of CDK4/Cyclin D1 was downloaded from previously reported work [25]. Later, the proteins were prepared by removing HETATM lines other than co-factors and unwanted water molecules from the structure. Along with this protonation, ionization, energy minimization, and hydrogen bonds were added. The CHARMm force field was applied for optimizing the geometry. The prepared protein was used for defining the binding site from the “Edit binding site” option from the receptor-ligand interaction toolbar. By using the bound ligand binding position, the active site was created and found to possess 9.16 A˚ radius [26]. Docking was carried out by all prepared ligands. Structure-based virtual screening was carried out by docking all prepared ligands with each of the protein structures at the defined active site using Libdock from the receptor-ligand interactions toolbar. According to the Libdock score, all the docked ligand poses were graded and grouped by names.

### 2.10. Statistical Analysis

All data were presented as mean ± SEM from three independent experiments (*n* = 3). Statistical significance of differences in drug-treated versus control cells was determined using the Student *t*-test. The minimal level of significance was *p* < 0.05. All the graphs were plotted using GraphPad 5.0 and Origin software 9.5.

## 3. Results

### 3.1. Lanatoside C Exhibits Cytotoxic Effects Only on Cancer Cells

**Figure 1:**
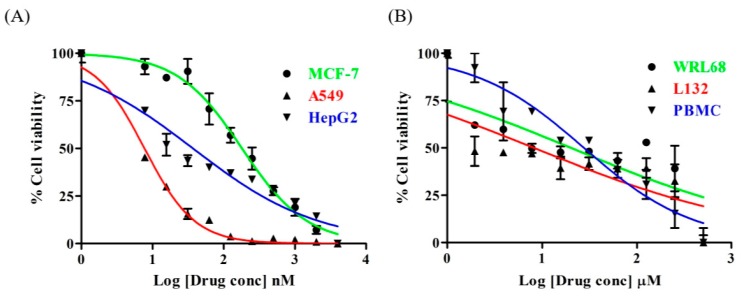
Lanatoside C effectively suppresses the growth of human cancer cell lines. Cell viability of (**A**) Lanatoside C in MCF-7, A549 and HepG2 cells in comparison with (**B**) L132, WRL68 cell lines and PBMCs.

In order to assess the cytotoxic effects of Lanatoside C in MCF-7, A549, and HepG2 cells, the cells were treated with a wide range of concentrations of Lanatoside C for 24 h and assessed for cytotoxic effects using MTT assay. Lanatoside C showed cell death in a dose-dependent manner. The IC_50_ values for Lanatoside C are 0.4 ± 0.1 µM, 56.49 ± 5.3 nM and 0.238 ± 0.16 µM for MCF-7, A549 and HepG2 cell, respectively (Figure 1A). The IC_50_ values were found to be related as A549 < HepG2 < MCF-7 cells. In order to estimate the cytotoxicity with respect to normal cells, L132 and WRL68 were treated with a wide range of concentrations (0.1–500 μM) for 24 h, and as expected we did not find any toxicity at the concentrations where it showed inhibition to cancer cell proliferation (Figure 1B). Further, we checked the toxicity in PBMC and observed that no toxicity was found up to 100 µM. MCF-7, A549, and HepG2 cells were treated with IC_50_ concentrations of Lanatoside C for 24 and 48 h. Morphological changes in the cells were observed and represented in microscopic images Figure S1.

### 3.2. Lanatoside C Treatment Induces DNA Damage in Cancer Cell Lines

Alkaline DNA damage version (pH > 13) was used to assess the DNA damage upon treating with Lanatoside C for 24 h period. We observed comets in all three cancer cell lines with high frequency after Lanatoside C treatment for 24 h in MCF-7, A549, and HepG2 while no obvious comets were found in the control cells. We observed comet tails and movement of the comet in treatments Figure S2, which was measured by using CASP lab software (Table 1), along with head and tail DNA percentages.

### 3.3. Lanatoside C Treatment Increases the Percentage of G2/GM and S Phase Cells in Cancer Cell Lines 

**Figure 2:**
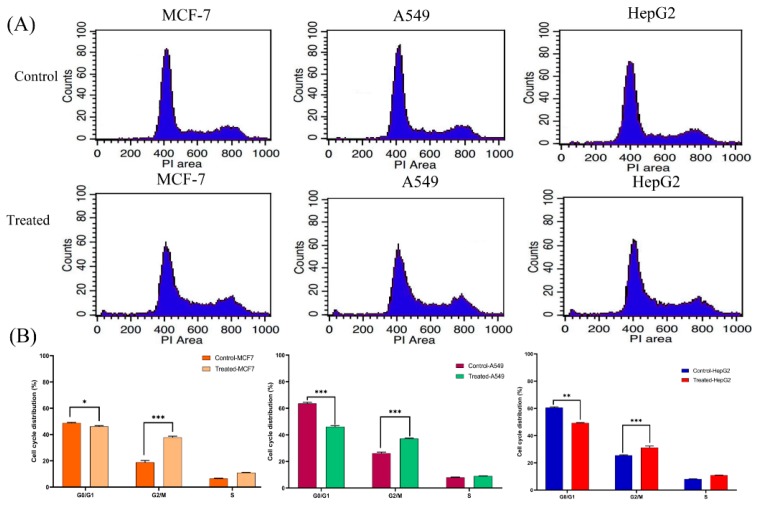
Lanatoside C induces cell cycle arrest at G2/M phase. (**A**) Controls and treatments of MCF-7, A549, and HepG2 with Lanatoside C and stained with propidium iodide and the changes in cell cycle distribution was analyzed by flow cytometry. (**B**) Quantitative analysis and representation of flow cytometry data.

MCF-7, A549, and HepG2 cells were treated with lethal doses of their IC_50_; namely, (MCF-7-1.2 μM), (A549-0.16 μM), and (HepG2-0.7 μM); all subsequent experiments were carried out with the above indicated concentrations. The obtained results suggest that Lanatoside C arrests the cell cycle at G2/M phase. Briefly, 24.7% of control MCF-7 cells was gated in to G2/M phase, whereas in treatment the percentage of cells was raised to 31.29%. A5459 cells also displayed a similar effect as that of MCF-7 cells, where the percentage of control and treatment cells in G2/M phase were 24.72% and 37.15%, respectively. Likewise, we checked for the role of Lanatoside C in HepG2 cell cycle progression and found a similar effect, i.e., 24.87% of control cells and 36.69% of treated cells in G2/M phase. This indicates that Lanatoside C arrested cell cycle at the G2/M phase in breast, lung, and liver cancer cells (Figure 2A,B). To further validate this observation, we probed for important proteins and genes responsible for this arrest. For this study, we checked the expressions of CDK6, Chk1, Chk2, and Cyclin D1 genes and proteins.

### 3.4. Lanatoside C Inhibits Expression of G2/M Cell Cycle Regulator, MAPK, and PI3K/AKT Pathway Genes

We further investigated the expressions of a few important genes involved in apoptosis, cell cycle arrest, and autophagic cell death using real-time PCR. Initially, we checked the expression of proto-oncogenes (*c-FOS, c-MYC,* and *c-JUN*) with Lanatoside C treatment for 24 h and found consistent downregulation in MCF-7, A549, and HepG2 cells. We also checked the expressions of some tumor suppressor genes (TSGs) (*JAK, PTEN,* and *p53*); upregulation of TSGs was identified in all Lanatoside C treated cancer cells. Next, we extended our study to inspect the expression of *p38MAPK*, *MEK1, MAPK24, P44,* and *SAPK/JNK* from the MAPK pathway. Lethal doses of Lanatoside C incubation for 24 h resulted in decreased expression levels of *p38MAPK, MEK1,* and *MAPK24* in all the studied cancer cells. However, cell-specific gene expressions were observed in *SAPK/JNK,* as it was overexpressed in MCF-7 and HepG2 cells, whereas it was found to be dysregulated in A549. Overexpression of *P44* was observed in MCF-7 and A549 cells, whereas it was found to be dysregulated in HepG2. Subsequently, we evaluated the effect of Lanatoside C in *p62, AKT, PI3K, mTOR, LC3*, *Beclin, Sestrin,* and *STAT3* from PI3K/AKT/mTOR signaling. *AKT, p62, PI3K, mTOR, LC3*, *Beclin,* and *Sestrin* genes displayed downregulation in Lanatoside C treated MCF-7, A549, and HepG2 cells. To evaluate the effect of Lanatoside C in G2/M phase arrest, we checked the expressions of checkpoint kinases (*CHK1* and *CHK2*) and cyclin-dependent kinases *(CDK6* and *Cyclin D1*) in MCF-7, A549, and HepG2 cells. A consistent downregulation of *CHK1, CHK2, CDK6,* and *Cyclin D1* was observed, which confirms that cell cycle arrest at the G2/GM phase could be substantial for Lanatoside C treatment to cancer cells. We further validated the role of Lanatoside C in attenuating the Wnt signaling pathway through the expressions of *GSK3α* and *β-catenin*. Lanatoside C at 24 h treatment inhibited the expressions of both Gsk3α and *β-catenin,* as well as the related downstream target genes such as *Cyclin D1* and *c-MYC*. Expressions of *NF-kB* and *MSK1* were also assessed with Lanatoside C treatment for 24 h and we found that both of these genes were highly expressed in Lanatoside C treated cancer cells.

### 3.5. Lanatoside C Down-Regulates BCL-2 and Up-Regulates BAX to Induce Apoptosis in Cancer Cell Lines

**Figure 3:**
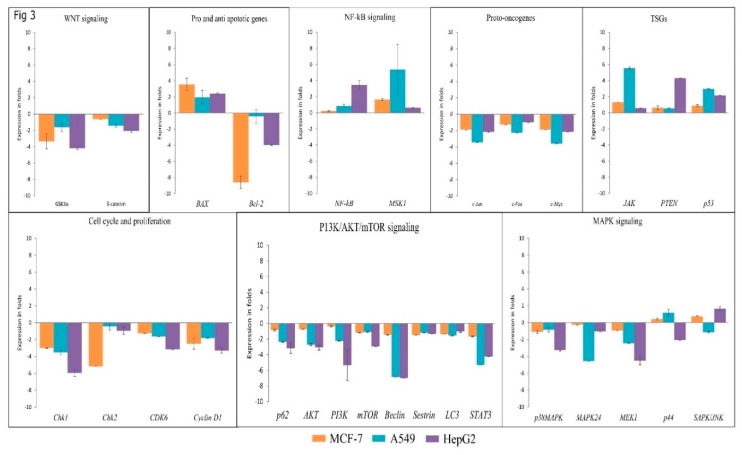
Gene expression analysis of various genes related to cell death and survival from various signaling pathways in Lanatoside C treated MCF-7, A549, and HepG2 cells, where GAPDH was used as an internal control. (i) Down-regulation of WNT signaling genes (GSK3α, and β-catenin). (ii) Expressions of pro and anti-apoptotic genes (BAX, and BCL-2). (iii) Expression significances on NF-kB signaling genes (NF-kB, and MSK1). (iv)Dysregulation of proto-oncogenes (c-FOS, C-MYC, and c-JUN). (v) Up-regulation of TSGs (JAK, PTEN, and P53). (vi) Downregulation of cell cycle regulating checkpoint and cyclin-dependent kinases (CHK1, CHK2, CDK6, and Cyclin D1). (vii) Genes that are involved in apoptosis and autophagy modulation from PI3K/AKT/mTOR signaling and their expressions (p62, AKT, PI3K, mTOR, Beclin, Sestrin, LC3, and STAT3). (viii) Gene expression studies of MAPK signaling (p38MAPK, MAPK24, MEK1, p44, and SAPK/JNK). All the expressions were analyzed with 2^−∆∆Ct^ method and the obtained results are statistically significant (*n* = 3 and *P* ≤ 0.001 and *P* ≤ 0.05).

Further, we checked the expression of one anti-apoptotic gene (*BCL-2*) and one proapoptotic gene (*BAX*) with Lanatoside C treatment and observed the dysregulation of the anti-apoptotic gene (*BCL-2*) and overexpression of the proapoptotic gene (*BAX*). The obtained expressions were normalized to the reference gene *GAPDH*, and Log_2_ values were taken for determining fold changes in expression (Figure 3).

### 3.6. Lanatoside C Down-Regulates Cell Cycle Checkpoint Protein’s Expression to Exhibit Growth Arrest in Cancer Cell Lines

**Figure 4:**
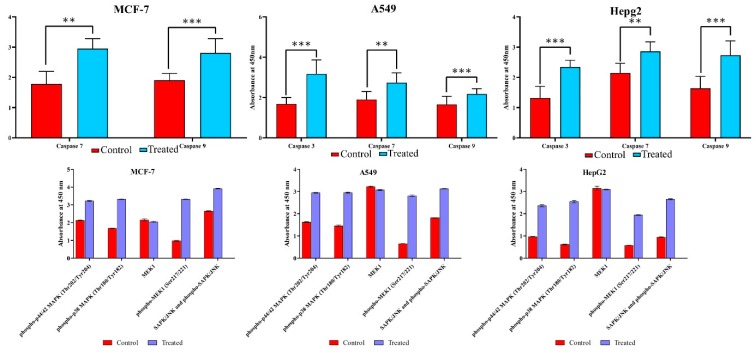
ELISA for the expression analysis of caspases and MAPK signaling pathway proteins. Over expressions of Caspase-3, 7, and 9 in Lanatoside C treated cells along with controls were observed. Upregulation of Phospho-P44/42 MAPK (Thr202/Tyr204), Phospho-p38 MAPK (Thr180/tyr182), MEK1, Phospho-MEK1 (Ser217/221), SAPK/JNK and Phospho-SAPK/JNK (Thr183/Tyr185) were assessed by ELISA in Lanatoside C treated MCF-7, A549, and HepG2 cells.

**Figure 5:**
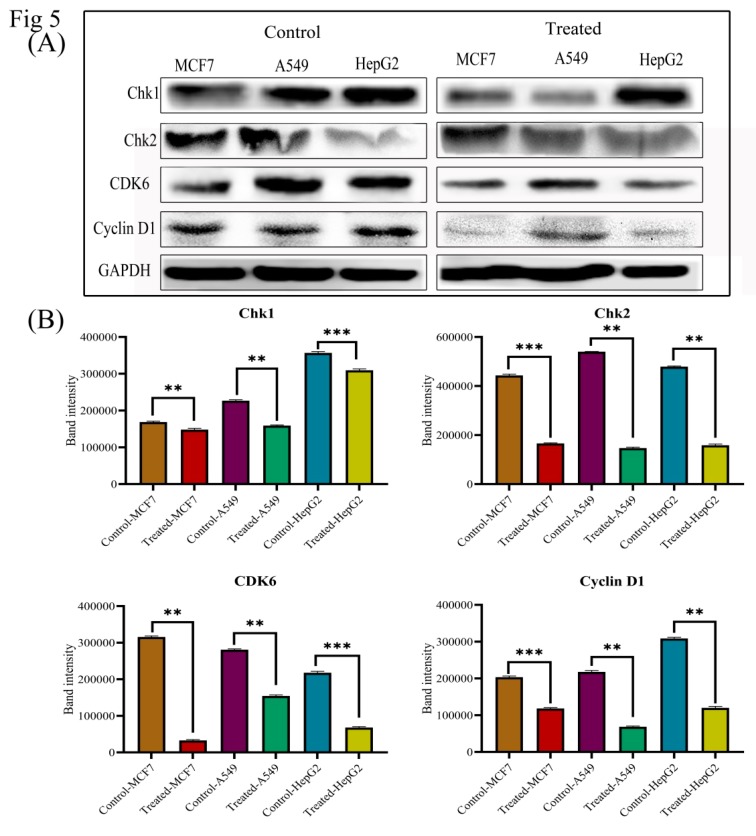
(**A**) Western blot expressions of target proteins with Lanatoside C treatment. Expression of cell cycle regulating proteins such as CHK1, CHK2, CDK6, and Cyclin D1 in three cancer cell lines. (**B**) Statistical analysis of expressions in Lanatoside C treated cancer cells. Blots were compared with that of GAPDH expression to compare equal loading of samples.

Drug-induced apoptosis in cancer cells is mainly mediated by two mechanisms, i.e., either mitochondrial (intrinsic) or death receptor (extrinsic) mechanism through Caspase activations [26]. In order to perceive whether Lanatoside C affects apoptosis in MCF-7, A549, and HepG2 cells, we performed ELISA with the total cell lysate for 24 h of incubation and found that the lethal doses of Lanatoside C treatment significantly increased the expression of Caspase 3, 7, and 9 in MCF-7, A549, and HepG2 cells compared to untreated control (Figure 4). The cell cycle arrest due to Lanatoside C was further evaluated by western blot by checking the expressions of checkpoint and cyclin-dependent kinases (CHK1, CHK2, CDK6, and Cyclin D1), and we observed that all these proteins were significantly suppressed by lethal dosage treatment with Lanatoside C for 24 h in MCF-7, A549, and HepG2 cells compared with untreated control (Figure 5).

### 3.7. Lanatoside C Inhibits MAPK/Wnt, JAK-STAT, and PI3K/AKT/mTOR Pathways

**Figure 6:**
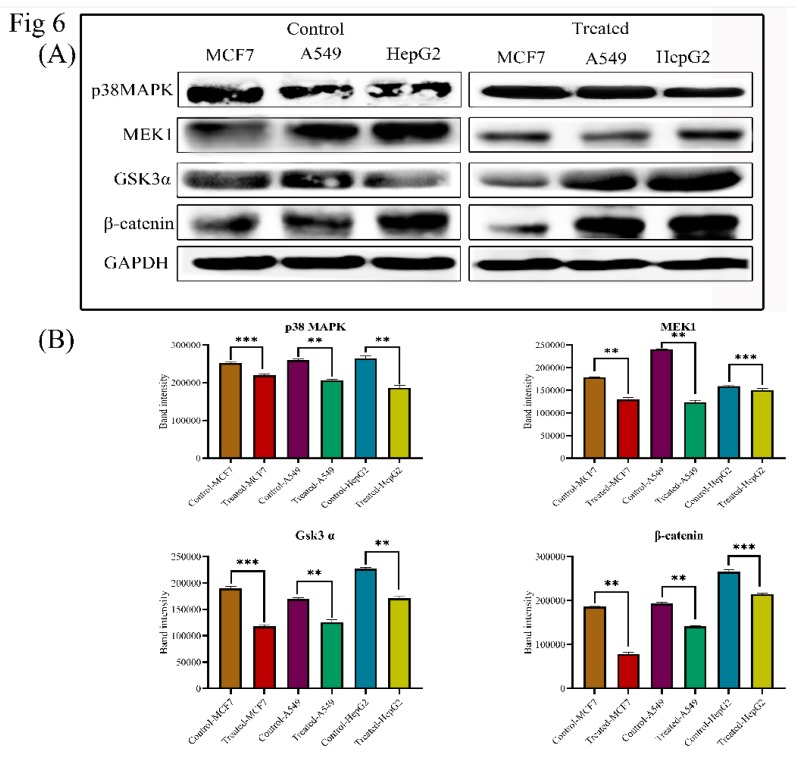
(**A**) Western blot and statistical analysis of proteins from MAPK and Wnt/β-catenin signaling. Consistent down regulation of p38MAPK and MEK1 was observed. Both Gsk3α and β-catenin are down regulated in Lanatoside C treated cancer cells. Blots were compared with that of GAPDH expression to compare equal loading of samples. (**B**) Band intensities of p38MAPK, MEK1, Gsk3α and β-catenin proteins.

In order to investigate the mechanism of Lanatoside C in inducing apoptosis, we attempted to check the expression of crucial proteins from MAPK signaling. Expressions of Phospho-P44/42 MAPK (Thr202/Tyr204), Phospho-p38 MAPK (Thr180/tyr182), MEK1, Phospho-MEK1 (Ser217/221), SAPK/JNK, and Phospho-SAPK/JNK (Thr183/Tyr185) were assessed in ELISA. We found a significant upregulation of all the phosphorylated proteins through ELISA and identified the downregulation of p38MAPK and MEK1 in Lanatoside C treatment by western blotting (Figure 4 and Figure 6). To examine these effects, we monitored the expression of the Wnt/β-catenin signaling pathway. Protein level expressions of GSK3α and β-catenin were obtained and we observed that both proteins were consistently downregulated in all the cancer cells treated with lethal concentrations of Lanatoside C (Figure 6). Downregulation of these proteins also resulted in the inhibited expression of downstream target proteins such as c-MYC and Cyclin D1 resulting in apoptosis and cell cycle arrest in Lanatoside C treated MCF-7, A549, and HepG2 cells.

**Figure 7:**
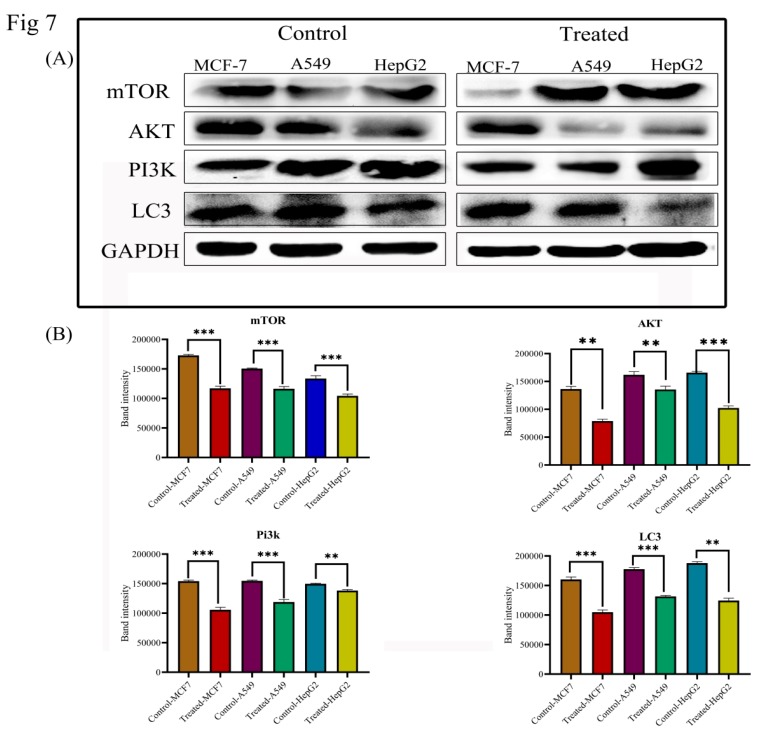
(**A**) Western blot and statistical analysis of proteins from PI3K/AKT/mTOR signaling. Down-regulation of AKT, mTOR, LC3, and PI3K was observed. Blots were compared with that of GAPDH expression to compare equal loading of samples. (**B**) Quantification of band intensities of AKT, mTOR, LC3 and PI3K in Lanatoside C treated cells in comparison with controls.

To determine whether Lanatoside C induces cell death through autophagy in MCF-7, A549, and HepG2 cells, we attempted to check the protein expressions of PI3K/AKT/mTOR signaling. Protein expressions of AKT, mTOR, PI3K, p62, LC3, and Beclin 1 were evaluated through western blotting (Figure 7). AKT, PI3K, mTOR, p62, LC3, and Beclin 1 showed consistent downregulation of gene expressions in Lanatoside C treated cancer cells compared to untreated cells.

### 3.8. Immunofluorescence Analysis Based Confirmation of Pathways Attenuated by Lanatoside C

**Figure 8:**
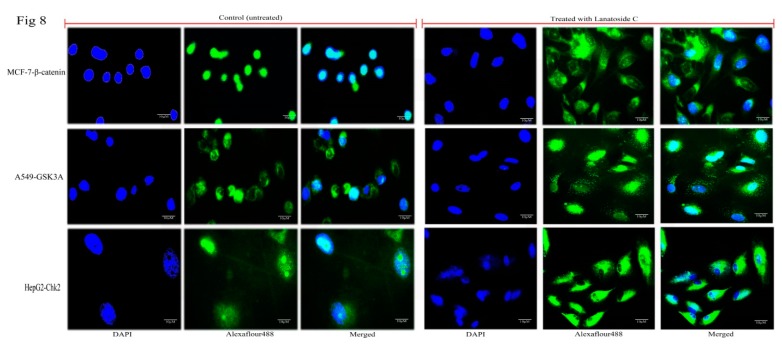
Immunofluorescence imaging of subcellular localization of target proteins in MCF-7 cells (β-catenin), A549 cells (Gsk3α) and HepG2 cells (CHK2). Scale bar 10 µM.

We next performed immunofluorescence studies to understand the underlying anticancer mechanism in Lanatoside C treated MCF-7, A549, and HepG2 cells compared to untreated cells (Figure 8). Further, we checked expressions of various proteins from different signaling pathways including MAPK signaling, Wnt/β-catenin signaling, and PI3K/AKT/mTOR signaling. Proteins involved in the cell cycle regulation (CHK1, CHK2, CDK6, and Cyclin D1), pro-apoptotic protein (BAX) and proto-oncogene (c-MYC) were also checked for their localization alterations. Proteins such as p38MAPK, MEK1, and SAPK/JNK were observed to change their localization in Lanatoside C treated cancer cells compared with controls. Next, we checked the localization of cell cycle regulating proteins such as CHK1, CHK2, Cyclin D1, and CDK6 in Lanatoside C treated cancer cells and observed the presence of proteins in the extracellular matrix. Furthermore, we tested the localization and expressions of Wnt/ β-catenin signaling pathway proteins leading to cell cycle arrest. GSK3α, β-catenin, and Cyclin D1 localizations were found to be positioned from the nucleus to the extracellular matrix and/or membrane compared to untreated cells. Along with that, we detected the localization of vital proteins involved in Autophagy inhibition from PI3K/AKT/mTOR signaling. All the microscopic observations are shown in Figures S3A–3D, 4A–4D, and 5A–5D. Illustrative images were obtained at 60× *g*.

### 3.9. Molecular Docking Analysis Shows Lanatoside C can Potentially Inhibit Multiple Cancer Targets

**Figure 9:**
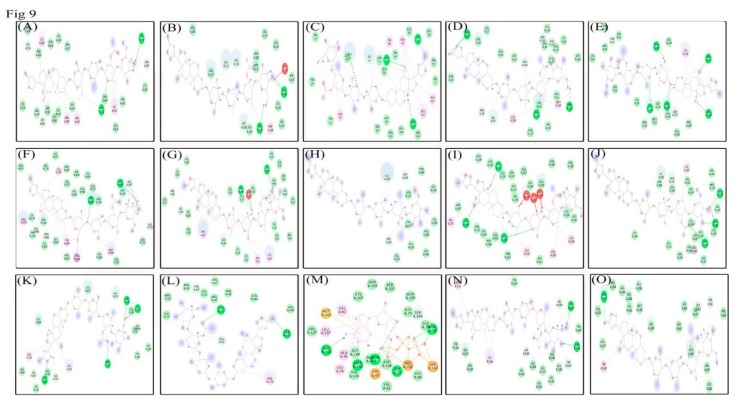
2D interactions of (**A**) STAT3, (**B**) BCL-2, (**C**) Cyclin D1, (**D**) p38, (**E**) NF-kB, (**F**) PARP, (**G**) CHK1, (**H**) AKT, (**I**) CHK2, (**J**) JNK, (**K**) PI3K, (**L**) CDK6, (**M**) MEK1, (**N**) mTOR, and (**O**) JAK with Lanatoside C.

Molecular docking studies were performed to identify protein-ligand relationships of Lanatoside C with various cancer therapeutic proteins. Binding with more hydrogen bonds was considered more stable, and the LibDock score was ranked based on the number of hydrogen bonds formed. Initially, we checked the role of Lanatoside C in tumor-associated proteins such as AKT, PI3K, and mTOR. Lanatoside C bound to all these therapeutic proteins with the highest Libdock scores and maximum hydrogen bond formations. AKT formed three hydrogen bonds with Lanatoside C at GLU323, ASP324, and ASP326 residues with a Libdock score of 51.2917. PI3K bound with Lanatoside C by forming seven hydrogen bonds at ILE703, ALA704, SER706, ARG707, SER753, LYS809, and LYS807 residues with 105 Libdock score. mTOR also showed strong binding with Lanatoside C by forming five hydrogen bonds with ASP2360, VAL2364, ASP2433, THR2434, and THR2436 residues with 83.6223 score. Cell cycle regulating proteins such as CHK1, CHK2, CDK6, and Cyclin D1 also showed good binding affinity with Lanatoside C. Chk1 and Chk2 bound with Lanatoside C and formed 5 and 6 hydrogen bonds, respectively. Residues GLU17, PRO98, ASP148, GLY150, and GLY204 interacted with Lanatoside C with a Libdock score of 161.897. From Chk2 residues, LEU226, CYS231, MET304, GLY307, GLU308, and LYS349 interacted with Lanatoside C with a score of 154.58. Cyclin-dependent kinases (CDK6 and Cyclin D1) also displayed a good binding affinity with 97.4948 and 172.401 Libdock scores respectively. CDK6 displayed the highest binding affinity by forming four hydrogen bonds with THR49, GLU51, GLU52, and GLN173 residues. Cyclin D1 formed six hydrogen bonds with Lanatoside C at GLU11, ILE12, GLY13, LYS35, HIS95, and ASP158 residues. Proteins from MAPK signaling also showed a good binding relationship with Lanatoside C. MEK1 showed the highest binding affinity with seven hydrogen bond formations with ALA76, GLY77, ASN78, LYS97, GLU144, MET146, and SER194 with a Libdock score of 137.537. Protein p38MAPK also displayed high binding affinity by forming eight hydrogen bonds with PRO29, VAL30, VAL38, ARG49, GLY110, ALA111, SER154, and ALA157 with a 140.632 score. SAPK/JNK made five hydrogen bonds with Lanatoside C at ILE108, ARG110, GLU179, MET181, and SER233 residues with a 102 Libdock score. Proteins from JAK-STAT signaling also displayed decent binding affinities with Lanatoside C. JAK showed worthy relation with Lanatoside C by making three hydrogen bonds with SER961, GLY962, and ARG1007 with a 87.0358 Libdock score. STAT3 also showed a worthy binding with Lanatoside C by developing five hydrogen bonds at GLY373, GLY421, GLN469, ASN472, and LYS551 with a 170.564 Libdock score. PARP also displayed good binding with Lanatoside C by forming 7 hydrogen bonds with residues, ALA755, ASP756, ALA880, PRO881, THR887, TYR907, and HIS937 residues through a 180.235 Libdock score. Similarly, NF-kB also exhibited good binding with Lanatoside C by forming seven hydrogen bonds with ARG33, THR52, ASN186, ARG187, GLU193, LYS195, and ASP217 residues with a 124.57 Libdock score. Anti-apoptotic protein (BCL-2) also displayed good binding with Lanatoside C by forming five hydrogen bonds at ASP108, GLU111, VAL130, GLU133, and ALA146 with a decent Libdock score of 116.154. Along with hydrogen bond-forming residues, several other residues interacted with Lanatoside C in 4 Å distance (Table 2), and the 2D interactions are represented in Figure 9. The protein-ligand docked complex is shown in Figure S6.

## 4. Discussion

Although many advanced therapies currently exist for cancer, it still remains one of the major causes of mortality in developed and developing countries all over the world [27,28,29,30,31,32,33]. Clinical and epidemiological studies have revealed that patients under treatment with digitalis have relatively fewer mortality rates due to cancer [34,35]. In-vitro and in-vivo experimentations from the last decade recognized that CGs demonstrate anticancer properties in many cancer cells [36]. Lanatoside C is one of the naturally-derived cardiac glycosides with multiple beneficial properties such as anti-viral, anti-inflammatory, and anticancer activities in some specific cancer cells [4,11]. In this study, we have identified the molecular mechanisms of anti-cancer activity of FDA approved cardiac glycoside Lanatoside C from *Digitalis ferruginea* in breast, lung, and liver cancer cells, along with their possible mechanisms of action. Potential interpretation for increased sensitivity towards Lanatoside C treatment is that it induces apoptosis, autophagic cell death, and cell-cycle arrest. Lanatoside C is more potent towards lung cancer cells with minimal inhibitory concentration (56.49 ± 5.3nM) compared to breast and liver cancer cells (0.4 ± 0.1µM and 0.238 ± 0.16µM, respectively) (Figure 1A). Hence, the cytotoxicity of Lanatoside C could be defined as lung > liver > breast cancers. No obvious toxicity has been observed in non-malignant cells such as those of the liver (WRL-68) and lung (L132). Lanatoside C possess absolutely no toxicity towards PBMCs even up to 250 µM concentrations. Cell cycle arrest at the G2/M phase was observed in all three cancer cells (Figure 2), and a similar phase arrest was reported earlier in Huh7 and mahlavu cells [37].

Here, we have checked the expressions of proto-oncogenes, tumor suppressor genes, anti-apoptotic proteins, and pro-apoptotic proteins to understand the preliminary mechanism of cell death in Lanatoside C treated cancer cells. Proto-oncogenes are known to perform physiological activities for maintaining cellular homeostasis for cell growth, differentiation, and proliferation [38]. Expressions of c-MYC, c-FOS and c-JUN were evaluated in the current study in MCF-7, A549, and HepG2 cells with Lanatoside C treatment. We observed the consistent downregulation of c-MYC, c-FOS, and c-JUN leading to apoptosis in Lanatoside C treatment. Recent studies have suggested that higher expression of the TSGs could lead to the suppression of cancer growth for many types of cancers [39]. We tested the expression of PTEN and p53 and identified that these genes were overexpressed in all the Lanatoside C treated cancer cells. STAT signaling also plays an important role in cancer disease progression and cell death, which makes STAT a possible cancer intervention candidate for the development of new drugs. Recent reports suggest that JAK-STAT is highly activated in all the tumors and is mainly responsible for physiological growth and cellular homeostasis [40,41]. Activated JAK could dysregulate the expression of STAT3 and can lead to the activation of multiple oncogenic signaling pathways [42]. Activated STAT3 is known to regulate tumor cell metastasis and angiogenesis; additionally, diverse tumor types showed persistent activation of STAT3 [43,44]. The present study revealed that the anticancer activity of Lanatoside C in various cancer cells could also be promoted through JAK-STAT signaling (Figure 3). These outcomes suggest that a comprehensive number of pathways are involved with the anticancer properties of Lanatoside C.

Drug-induced apoptosis in cancer cells is mainly mediated by a mitochondrial (intrinsic) or death receptor (extrinsic) mechanism through Caspase activations [45]. The activated gatekeeper Caspase 9 initiates the mitochondrial (intrinsic) apoptosis by activating Caspase 3 and 7 as observed in the present study (Figure 4). Also, we have checked the expression ratio of anti and pro-apoptotic genes *BCL-2* and *BAX*. Increased expression of BAX was observed in the current study, which is reported to terminate the mitochondrial membrane potential thereby causing apoptosis. DNA damage induced by anticancer agents can also result in cell cycle arrest at the G2/M phase through TP53 dependent and independent mechanisms to block the cells’ entry into mitosis [46]. We checked the expression of checkpoint kinase genes such as *CHK1* and *CHK2* in Lanatoside C treated cancer cells. Overexpression of checkpoint kinases was reported in most of the cancer cells and the downregulation of this could lead to cell death by cell cycle arrest at the G2/M phase [47]. One such mechanism was also evaluated in the present study and we identified the significant downregulation of checkpoint kinases in all Lanatoside C treated cancer cells. On the other hand, overexpression of cyclin-dependent kinases (CDK6 and Cyclin D1) was observed in untreated breast, lung, and liver cancer cells in the present study, corroborating previous reports [48]. Interestingly, Lanatoside C treatment to these cancer cells resulted in the downregulation of cyclin-dependent kinases (Figure 5).

MAPK pathway plays a crucial role in cancer disease progression and apoptosis [49]. A variety of signals such as chemotherapeutic drugs, ultraviolet radiations, and tumor necrosis factors can activate the crucial JNK and ERK1/2 from the MAPK signaling, which is a key signaling protein in mammalian mitogen signaling [50,51,52]. Activated JNK and ERK1/2 could lead to cell cycle arrest at the G2/M phase [53], which is also witnessed in our study. The role of p38MAPK and MEK1 was diverse in the cancer cells, as they can promote or inhibit cell survival [54]. In the present study, we showed that Lanatoside C at its lethal doses can alter the mechanism of MAPK signaling and inhibits p38MAPK, MEK1, and SAPK/JNK (Figure 4 and Figure 6). Taken together, our results suggest that MAPK signaling was also involved in the anti-cancer effect induced by Lanatoside C on breast, lung, and liver cancer cells.

Wnt/β-catenin signaling plays an essential role in embryonic development, stem cell regeneration, and tumorigenesis [55,56]. It has been reported that the Wnt/β-catenin pathway abnormally activates in several types of tumors [57]. Lanatoside C displayed a potent inhibitory effect on key target proteins of Wnt signaling, i.e., GSK-3α and β-catenin in Lanatoside C treated breast, lung, and liver cancer cells. Dysregulation of this pathway leads to the activation of proto-oncogenes (c-MYC) and cyclin-dependent kinases (Cyclin D1), which eventually leads to cancer cell proliferation [58,59]. Obtained results disclosed that Lanatoside C inhibits Wnt/β-catenin signaling, along with Cyclin D1 and c-MYC expression in breast, lung, and liver cancers. Taken together, our data demonstrate the effect of Lanatoside C in modulating Wnt/β-catenin signaling, which could enhance the importance of inhibitors for β-catenin and its transcriptional targets including c-MYC, Cyclin D1, and CDK6 may serve as potential targets for developing novel therapeutic drugs against human cancers (Figure 6).

Autophagy through PI3K/AKT/mTOR pathway encourages cell growth by degrading the damaged proteins and other organelles in the cell [60,61,62,63]. Thus, activation of the PI3K/AKT/mTOR pathway plays a vital role in cancer disease progression, protein synthesis, and is recognized as one of the key targets for novel treatment approaches [64,65]. Inhibitors of the PI3K/AKT/mTOR pathway have been recognized as novel drugs for anticancer therapy [66]. Inhibitors of mTOR and PI3K through various stress conditions by chemotherapeutic drugs lead to the activation of AKT, which directly inhibits mTOR through several regulators. We have checked the role of Lanatoside C in PI3K/AKT/mTOR pathway to identify its role in autophagy, as recent reports suggested that CGs (Cerberin) cause apoptosis and autophagy inhibition through PI3K/AKT/mTOR signaling [67]. We have identified the dysregulation of AKT, mTOR, p62, LC3, and Beclin 1, which has led to the initiation of cell death by Lanatoside C in breast, lung, and liver cancer cells. Hence, the results suggested that Lanatoside C can act as an inhibitor for PI3K/AKT/mTOR pathway to induce apoptosis, cell cycle arrest, and inhibits autophagy in breast, lung, and liver cancer cells (Figure 7). The summary of all the genes and protein expressions from this study are listed in Table S2.

Understanding the molecular interactions between the target protein and ligand could lead to the discovery of novel drugs for many diseases. Hence, we checked the molecular interaction between tumor-associated proteins such as AKT, PI3K, mTOR, and cell cycle regulating proteins such as CHK1, CHK2, CDK6, Cyclin D1, and proteins from MAPK signaling including MEK1, p38MAPK, and SAPK/JNK. Crucial proteins from JAK-STAT signaling such as JAK and STAT3 also showed binding affinity with Lanatoside C. Anti-apoptotic protein (BCL-2) and PARP also showed important binding affinity with the highest Libdock scores (Table 2).

## 5. Conclusions

The present study demonstrates the cytotoxic activity of Lanatoside C against different types of cancer, including breast, lung, and liver cells. Cell cycle arrest at the G2/M phase was also witnessed in this study through FACS, and this effect was further authenticated by identifying the deregulation of checkpoint kinases and cyclin-dependent kinases. Caspase-mediated intrinsic apoptosis was also detected along with the upregulation of pro-apoptotic genes and downregulation of anti-apoptotic genes. Apoptosis through attenuation of MAPK signaling was observed in Lanatoside C treated cancer cells by noticing the downregulation of p38MAPK, MEK1 and SAPK/JNK. We also identified that JAK-STAT signaling was also involved in the apoptotic mechanism of Lanatoside C. Autophagic cell death through PI3K/AKT/mTOR signaling was identified by observing the downregulation of PI3K, mTOR, LC3, Beclin 1, and p62. Apoptosis through Wnt/β-catenin signaling as shown in this study can motivate the design of unique treatment strategies by the identification of new chemotherapeutic drugs. To the best of our knowledge, this is the first report on Lanatoside C as a potent cytotoxic agent exhibiting novel mechanisms of action for its potential use in anticancer therapy against breast, lung, and liver cancer cells.

## Figures and Tables

**Table 1:** Distance of comets travelled with and without Lanatoside C for 24 h with IC_50_ concentrations.

Cells	Total Length of Comet	Length of Head	Length of Tail	Head DNA (%)	Tail DNA (%)	Tail Movement	Overall Tail Movement (OTM)
MCF7-control	62 ± 5	45 ± 3	14 ± 6	81 ± 6	18 ± 5	6. ± 2	7 ± 1
MCF7-Treated	252 ± 13	89 ± 6	162 ± 16	19 ± 5	80 ± 13	427 ± 36	261 ± 22
A549-control	112 ± 9	94 ± 6	18 ± 4	92 ± 8	9 ± 4	3 ± 1	6 ± 3
A549-Treated	321 ± 14	69 ± 5	251 ± 13	18 ± 2	81 ± 13	416 ± 42	221 ± 23
HepG2-control	132 ± 8	86 ± 7	46 ± 6	82 ± 12	17 ± 3	9 ± 4	64 ± 1
HepG2-Treated	362 ± 9	98 ± 6	264 ± 19	11 ± 2	88 ± 12	361 ± 41	112 ± 18

**Table 2:** Ligand interactions of Lanatoside C with various cell signaling proteins from different pathways and the residues that are forming hydrogen bonds along with amino acids at 4 Å distance.

S.no	PDB ID	Libdock Score	No. of H Bonds	Interacting Residues
1.	1BG1	170.564	5	**H bonds:** GLY373, GLY421, GLN469, ASN472, LYS551. **Interacting residues:** ASP369, LEU378, ARG379, GLY380, SER381, ARG382, LYS383, GLU415, GLN416, ARG417, CYS418, ASN420, GLY422, ARG423, ILE431, VAL432, THR433, ASN472, CYS550.
2.	1OVE	140.632	8	**H bonds:** PRO29, VAL30, VAL38, ARG49, GLY110, ALA111, SER154, ALA157. **Interacting residues:** ASN26, LEU27, SER28, GLY31, ALA40, ALA51, LYS53, HIS107, LEU108, MET109, ASP112, ASN115, ASN155, LEU156, LEU167, ASP168.
3.	1VKX	124.57	7	**H bonds:** ARG33, THR52, ASN186, ARG187, GLU193, LYS195, ASP217. **Interacting residues:** LYS28, ARG30, GLY31, MET32, PRO47, SER51, ASP53, LYS56, THR57, ALA188, PRO189, ALA192, LEU194, LYS218.
4.	1WOK	180.235	7	**H bonds:** ALA755, ASP756, ALA880, PRO881, THR887, TYR907, HIS937. **Interacting residues:** TYR710, ASN754, GLN759, GLU763, ASP766, LEU769, ASP770, HIS862, SER864, ASN868, ILE872, GLN875, LEU877, ARG878, ILE879, GLY888, TYR889, MET890, PHE891, GLY894, ILE895, TYR896, LYS903, ALA935, GLU988.
5.	2CBZ	102	4	**H bonds:** GLN713, GLN714, TRP716, GLN718. **Interacting residues:** TYR710, PRO712, PHE728, SER689.
6.	2E9P	161.897	5	**H bonds:** GLU17, PRO98, ASP148, GLY150, GLY204. **Interacting residues:** GLY18, ALA19, TYR20, LYS38, MET42, LYS43, GLU50, ASN51, ILE52, LYS54, GLU55, GLU91, PHE93, ILE96, GLU97, PRO98, LYS132, GLU134, ASN135, LEU151, ALA200, GLU205, LEU206.
7.	2JDO	51.2917	3	**H bonds:** GLU323, ASP324, ASP326. **Interacting residues:** THR306, GLU320, VAL321, ASN325, TYR327, GLY328, ASP388, PRO389, LYS390.
8.	CDK4 or CYCLIN D1	172.401	6	**H bonds:** GLU11, ILE12, GLY13, LYS35, HIS95, ASP158. **Interacting residues:** ALA10, VAL14, THR19, VAL20, TYR21, ALA33, LYS35, GLU56, VAL72, PHE93, GLU94, VAL96, ASP97, GLN98, ASP99, ARG101, THR102, GLU144, ASN145, LEU147.
9	2O21	116.154	5	**H bonds:** ASP108, GLU111, VAL130, GLU133, ALA146. **Interacting residues:** PHE101, TYR105, ARG106, ARG107, PHE109, MET112, VAL131, LEU134, PHE147, GLU149, PHE150, VAL153.
10	3ALN	102	5	**H bonds:** ILE108, ARG110, GLU179, MET181, SER233. **Interacting residues:** GLY109, GLY111, VAL116, ALA129, VAL162, MET178, LEU180, SER182, THR183, SER184, ASP186, LYS187, LYS190, ASN234, LEU236, CYS246, ASP247.
11	3EYG	87.0358	3	**H bonds:** SER961, GLY962, ARG1007. **Interacting residues:** ARG879, ASP880, LEU881, PRO960, SER963, LYS965, GLU966, LYS970, ALA1005, ALA1006, TRP1047, GLU1073, SER1080, SER1083, PRO1084, MET1085, ALA1086.
12	3L54	105	7	**H bonds:** ILE703, ALA704, SER706, ARG707, SER753, LYS809, LUY807. **Interacting residues:** GLY159, TYR160, GLN705, GLN710, LYS750, ALA754, GLU755, LYS756, LYS808, ASP874, LYS875.
13	3NUP	97.4948	4	**H bonds:** THR49, GLU51, GLU52, GLN173. **Interacting residues:** ARG46, VAL47, GLN48, GLY50, GLY53, MET54, PRO55, PHE172, GLN193, ALA248.
14	3VVH	137.537	8	**H bonds:** ALA76, GLY77, ASN78, LYS97, GLU144, MET146, SER194. **Interacting residues:** LEU74, GLY75, GLY79, GLY80, VAL81, VAL82, ALA95, VAL127, MET143, HIS145, GLY149, SER150, GLN153, LYS192, ASN195, LEU197, CYS207, ASP208.
15	4JSP	83.6223	5	**H bonds:** ASP2360, VAL2364, ASP2433, THR2434, THR2436. **Interacting residues:** PRO2116, THR2164, SER2165, LYS2166, ARG2168, ASP2338, HIS2340, ALA2365, THR2367, ARG2368, GLU2369, LYS2370, GLU2373, TYR2542.

## Supplementary Materials

The following are available online at https://www.mdpi.com/2218-273X/9/12/792/s1. In the Supplementary information file, Figures S1–S6 and Tables S1 and S2 can be found.
