# Supplementary material for: Lanatoside C Induces G2/M Cell Cycle Arrest and Suppresses Cancer Cell Growth by Attenuating MAPK, Wnt, JAK-STAT, and PI3K/AKT/mTOR Signaling Pathways
Source: Biomolecules. 2019 Nov 27;9(12):792. doi: 10.3390/biom9120792 (PMC6995510; doi:10.3390/biom9120792)
Supplement: Supplementary file 1 [file biomolecules-09-00792-s001.pdf]

Supplementary Data

Lanatoside C induces G2/M cell cycle arrest and suppresses cancer cell growth by attenuating MAPK, Wnt, JAK-STAT, and PI3K/AKT/mTOR signaling pathways

Dhanasekhar Reddy[1], Ranjith Kumavath[1*], Preetam Ghosh[2] and Debmalya Barh[3]

Gene	Forward primer	Reverse primer
Bcl-2	TTGTGGCCTTCTTTGAGTTCGGTG	GGTGCCGGTTCAGGTACTCAGTCA
Bax	CCTGTGCACCAAGGTGCCGGAACT	CCACCCTGGTCTTGGATCCAGCCC
CDK6	GGATAAAGTTCCAGAGCCTGGAG	GCGATGCACTACTCGGTGTGAA
Chk1	TTGGCTTCCTGCCACATGAT	TTGCAGTTTGCAGGACAGGA
Chk2	AGTGGTGGGGAATAAACGCC	TCTGGCTTTAAGTCACGGTGTA
p53	CAGCACATGACGGAGGTTGT	TCATCCAAATACTCCACACGC
MEK1	TGAGAGCGACGGTTCTCTACT	CACAATCAGAGTGTCCTGTTGTT
p44	ACTATGTCCGAAGCAAGGATTTC	CGCCCACTGATAATCTCTGGAG
p38 α	AACCTGTCTCCAGTGGGCTCT	CGTAACCCCGTTTTTGTGTCA
SAPK/JNK	GGGTATGCCCAAGAGGACAGA	GTGTTGGAAAAGTGCGCTGG
NF-kβ -P65	GTCAAAAACGCCACCTCTCAA	CTCGCATGGAATTTGGAACCG
AKT	CCTCCACGACATCGCACTG	TCACAAAGAGCCCTCCATTATCA
STAT3	CAGCAGCTTGACACACGGTA	AAACACCAAAGTGGCATGTGA
JAK	GCCAACGAGGATCTTCGAGC	CTTCTCGCGTTCCACTTTGC
p62	GCACCCCAATGTGATCTGC	CGCTACACAAGTCGTAGTCTGG
mTOR	ATGCAGCTGTCCTGGTTCTC	AATCAGACAGGCACGAAGGG
PI3K	CCACGACCATCATCAGGTGAA	CCTCACGGAGGCATTCTAAAGT
β-catenin	AGCTTCCAGACACGCTATCAT	CGGTACAACGAGCTGTTTCTAC
c-Myc	ATGGCCCATTACAAAGCCG	TTTCTGGAGTAGCAGCTCCTAA
Cyclin D1	GCTGCGAAGTGGAAACCATC	CCTCCTTCTGCACACATTTGAA
c-Jun	TGACTGCAAAGATGGAAACG	CAGGGTCATGCTCTGTTTCA
c-Fos	AAGGGAAAGGAATAAGATGGCT	GCAAAGCAGACTTCTCATCT
LC3	GGAGAATCCGAAGGGAAAG	TTGAGCTGTAAGCGCCTTCTA
Beclin 1	CTGGTAGAAGATAAAACCCGGTG	AGGTAGAGCGTGGACTATCCG
Sestrin 1	TGCTTTGGGCCGTTTGGATAA	TGTAGTGACGATAATGTAGGGGT
MAPK24 (MKK4)	GACGAGGAGCTTATGGTTCTGT	TTTTCATCCACTGTTGACCGAA
PTEN	AGGGACGAACTGGTGTAATGA	CTGGTCCTTACTTCCCCATAGAA
Msk1	CAACAATCGTTCAAAAGGCCAA	CGACTGCCTAATGTGTTCCAG
Gsk3A	GTGCCCGAGACAGTGTACC	ACACCTTGACATAGAGGATAGGG
GAPDH	AACGGGAAGCTTGTCATCAATGGAAA	GCATCAGCAGAGGGGGCAGAG

Supplementary Table 1: List of primers used in this study.

S.no	Gene/protein	Expressions (Up/Down)		
		MCF-7	A549	HepG2
1	c-Fos	↓	↓	↓
2	c-Myc	↓	↓	↓
3	c-Jun	↓	↓	↓
4	Chk1	↓	↓	↓
5	Chk2	↓	↓	↓
6	CDK6	↓	↓	↓
7	Cyclin D1	↓	↓	↓
8	MAPK24	↓	↓	↓
9	MEK1	↓	↓	↓
10	p38MAPK	↓	↓	↓
11	p62	↓	↓	↓
12	PI3K	↓	↓	↓
13	AKT	↓	↓	↓
14	mTOR	↓	↓	↓
15	Beclin	↓	↓	↓
16	LC3	↓	↓	↓
17	Sestrin	↓	↓	↓
18	Bcl-2	↓	↓	↓
19	Gsk3α	↓	↓	↓
20	β-catenin	↓	↓	↓
21	NF-kB	↑	↑	↑
22	Msk1	↑	↑	↑
23	Bax	↑	↑	↑
24	STAT3	↑	↓	↑
25	p53	↓	↓	↑
26	p44	↑	↑	↓
27	JAK	↑	↑	↓
28	SAPK/JNK	↑	↓	↑
29	PTEN	↑	↑	↓

↑ **Upregulation** ↓ **Downregulation**

Supplementary Table 2: Summary of identified gene/protein expressions in this study with Lanatoside C treatment.

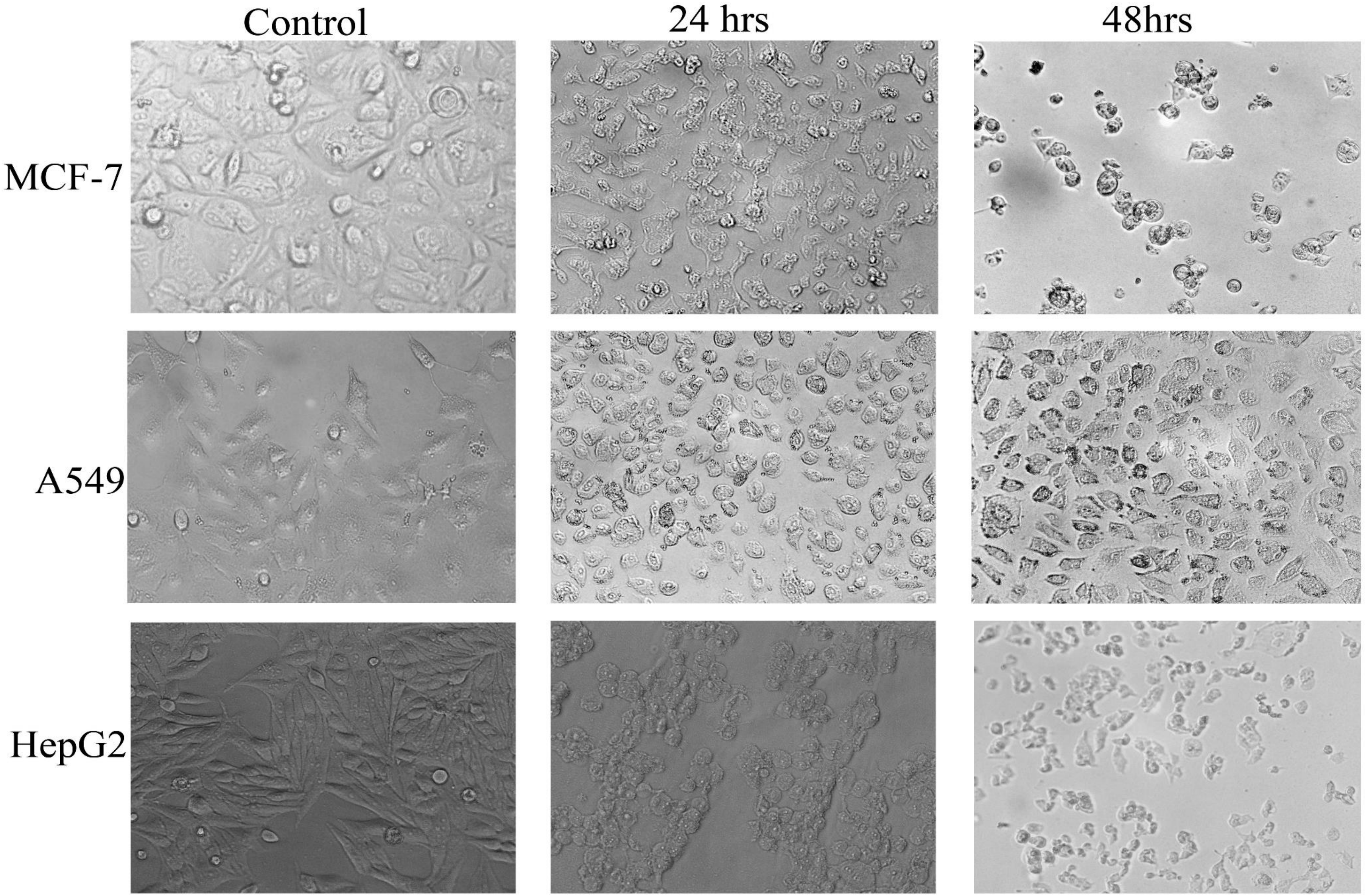

Supplementary Fig. 1: MCF-7, A549 and Hepg2 cells were treated with Lanatoside C for 24 or 48 h. Morphological changes in the cells were observed. Representative images were obtained at 40× magnification. Scale bar: 50 µm.

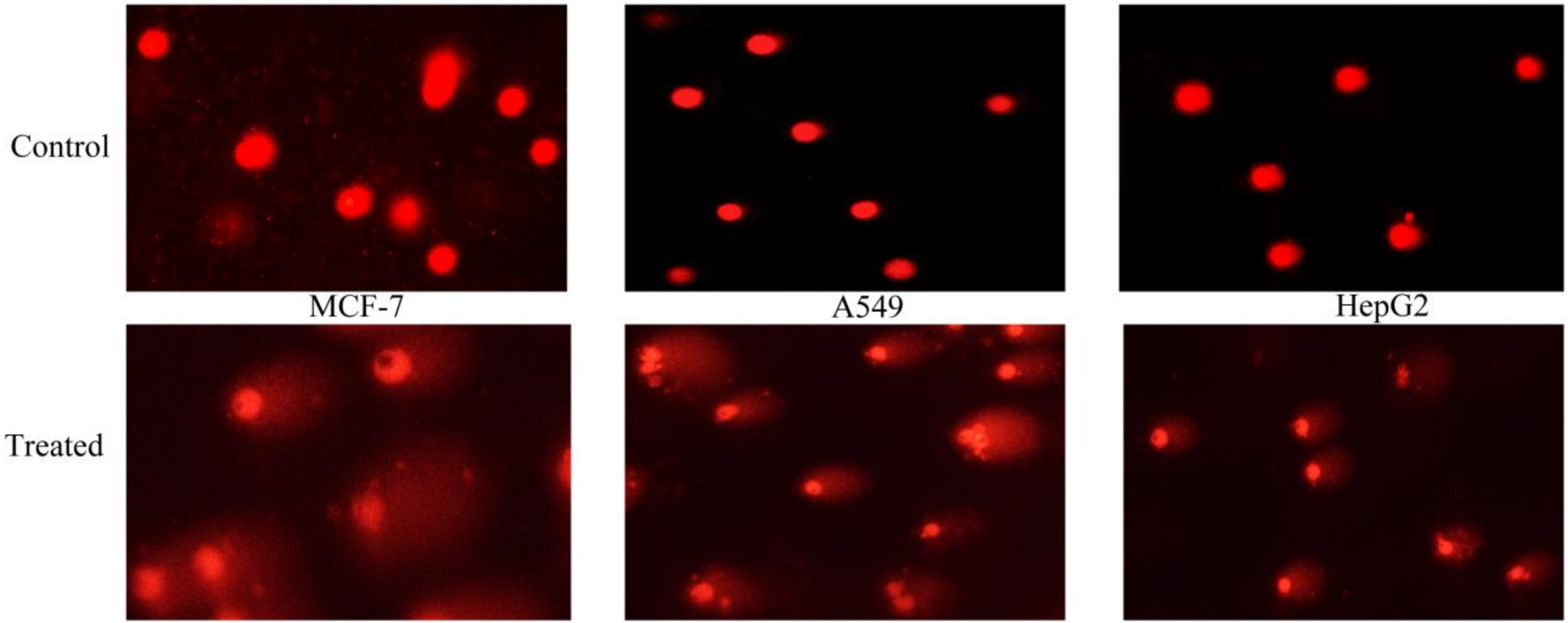

Supplementary Fig. 2: Lanatoside C treated cells showing DNA damage in MCF-7 , A549 , and HepG2 cells compared to untreated control cells..

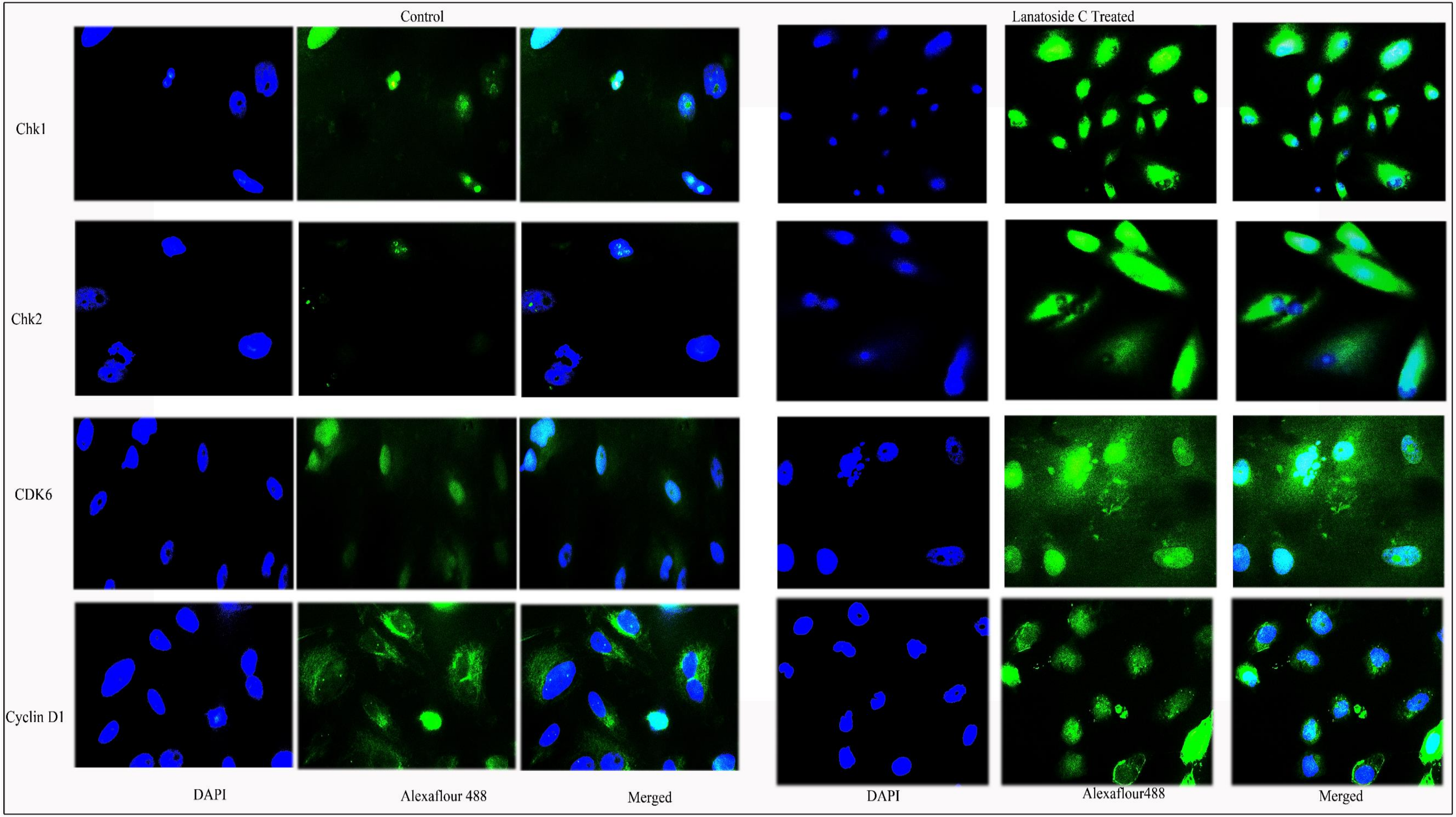

Supplementary Fig. 3A: Immunofluorescence imaging for the analysis of protein localisations of Chk1, Chk2, CDK6 and Cyclin D1 in Lanatoside C induced MCF-7 cells.

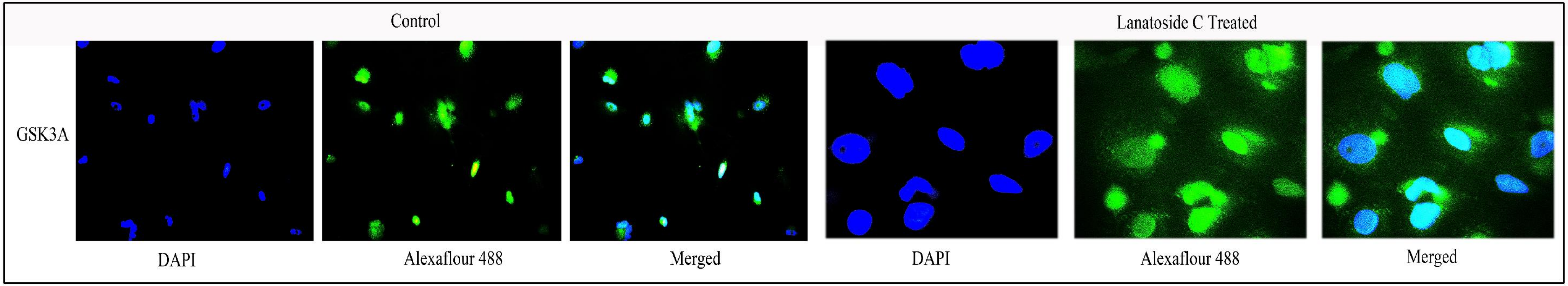

Supplementary Fig. 3B: Immunofluorescence imaging for the analysis of protein localisation of Gsk3α in Lanatoside C induced MCF-7 cells.

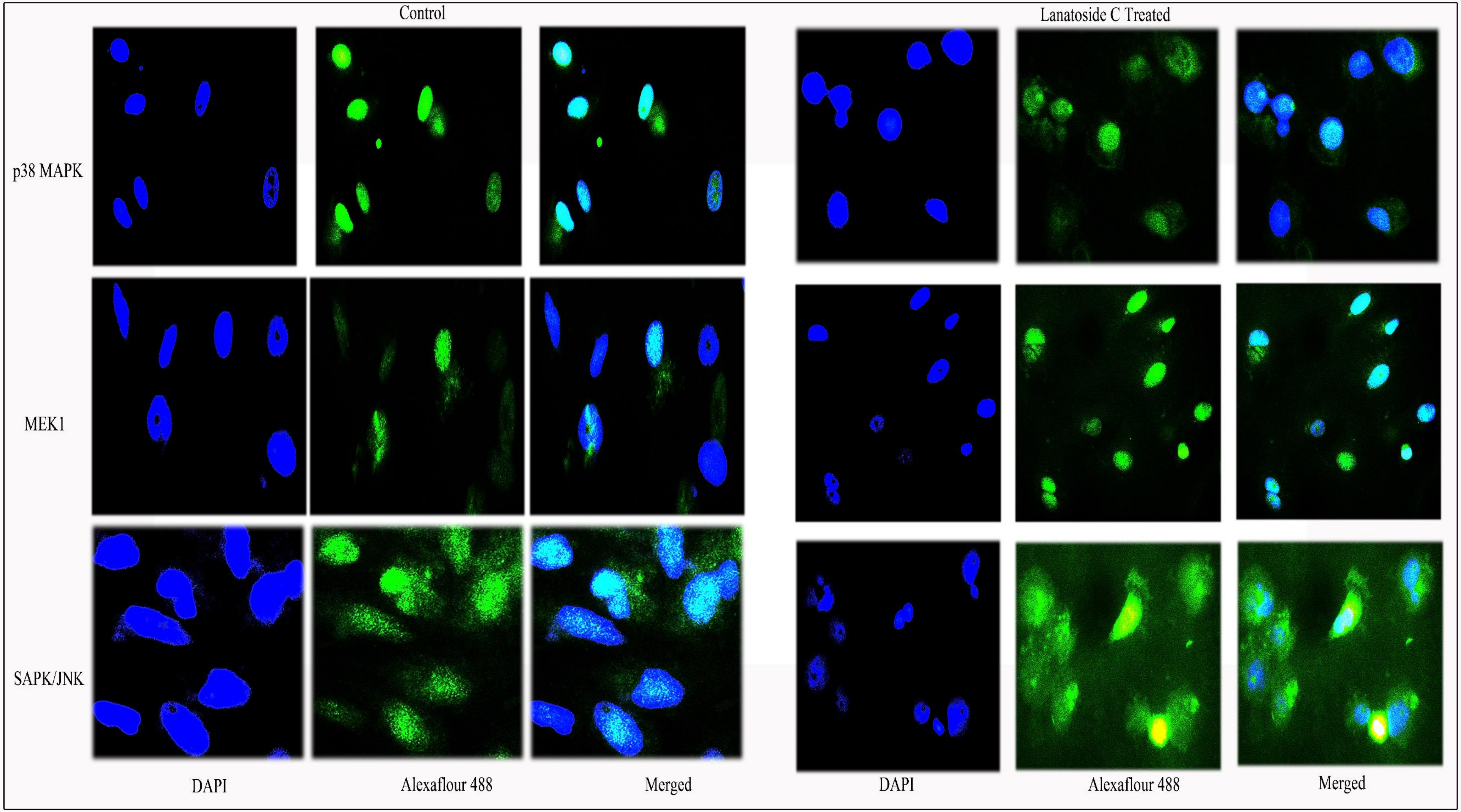

Supplementary Fig. 3C: Immunofluorescence imaging for the analysis of protein localisations of p38MAPK, MEK1and SAPK/JNK in Lanatoside C induced MCF-7 cells.

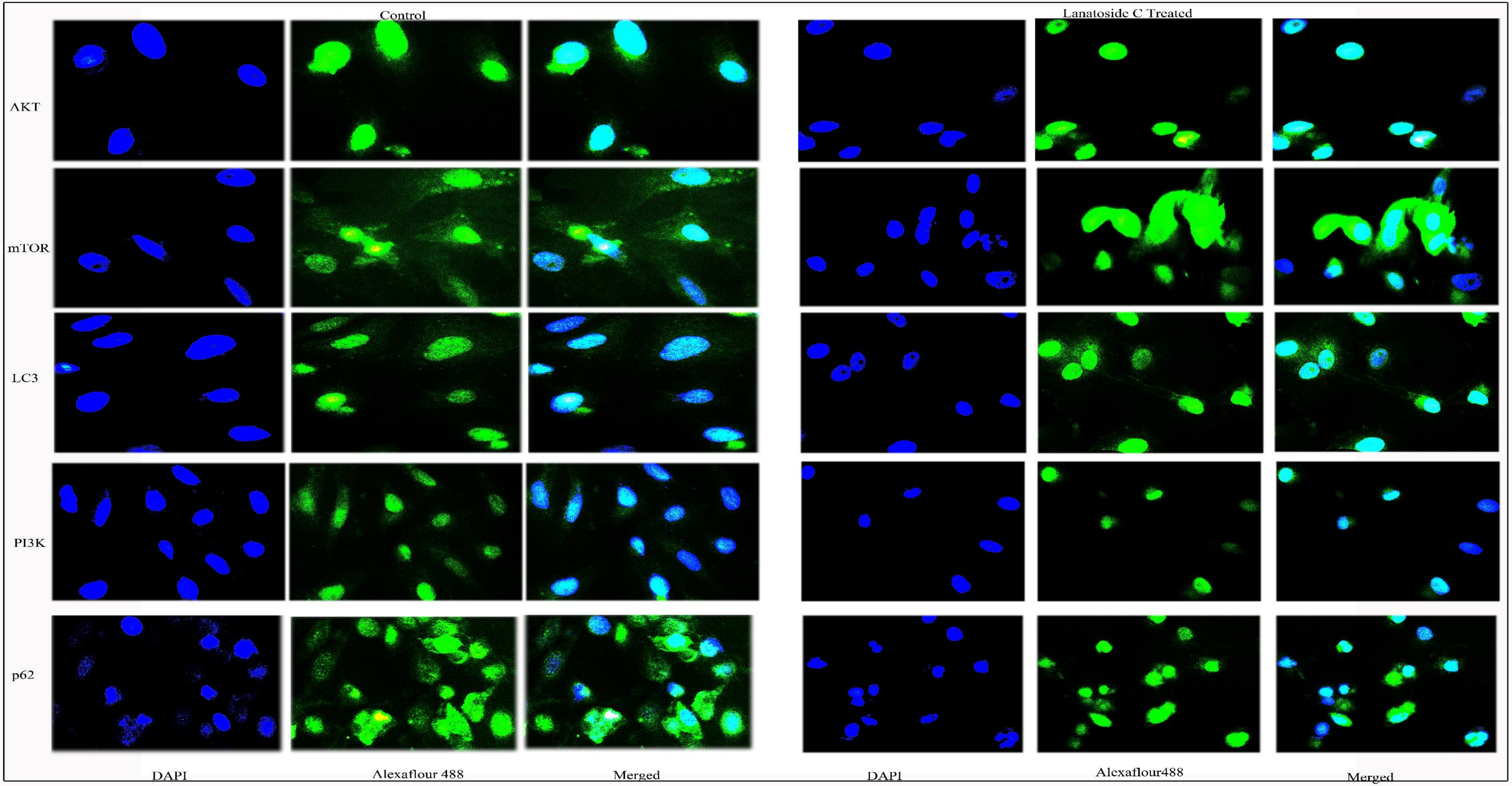

Supplementary Fig. 3D: Immunofluorescence imaging for the analysis of protein localisations in AKT, mTOR, LC3, PI3K and p62 in Lanatoside C induced MCF-7 cells.

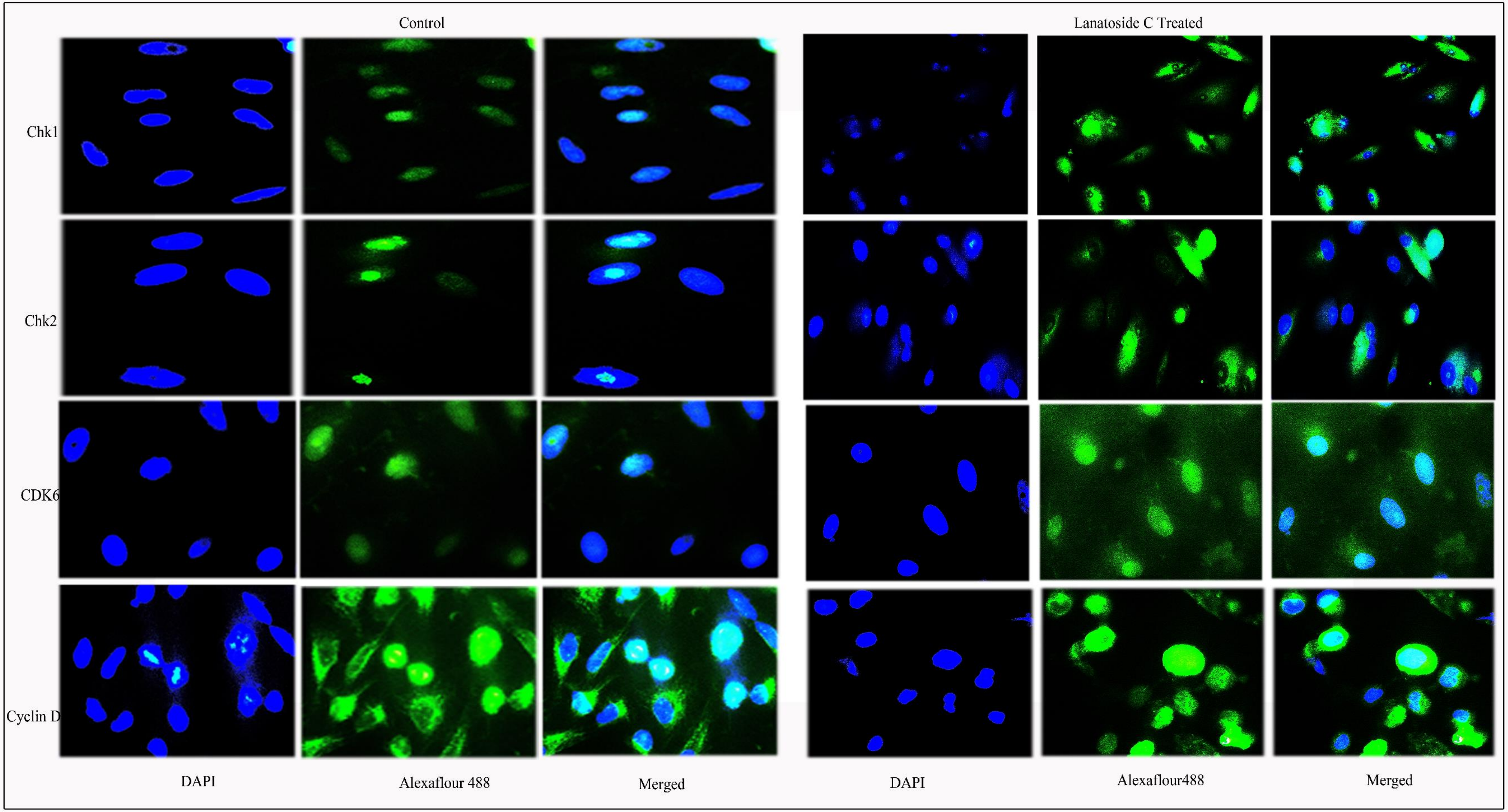

Supplementary Fig. 4A: Immunofluorescence imaging for the analysis of protein localisations of Chk1, Chk2, CDK6 and Cyclin D1 in Lanatoside C induced A549 cells.

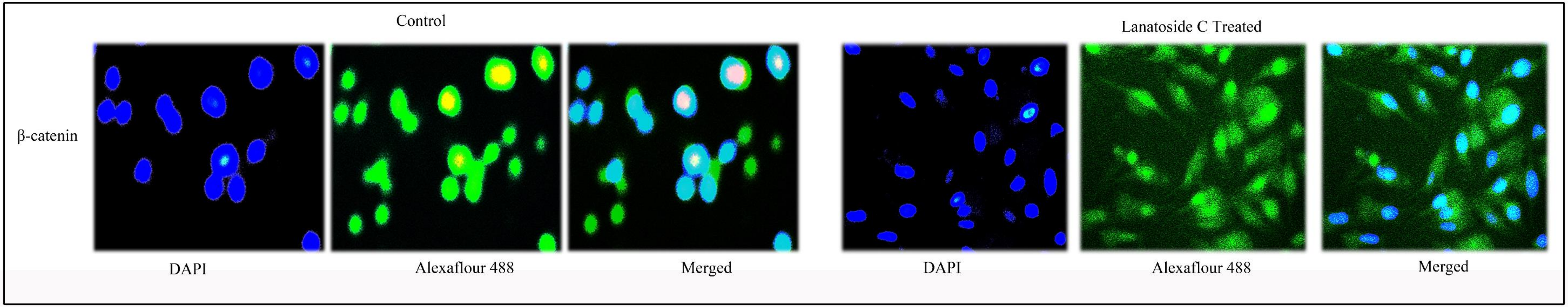

Supplementary Fig. 4B: Immunofluorescence imaging for the analysis of protein localisation of β-catenin in Lanatoside C induced A549 cells.

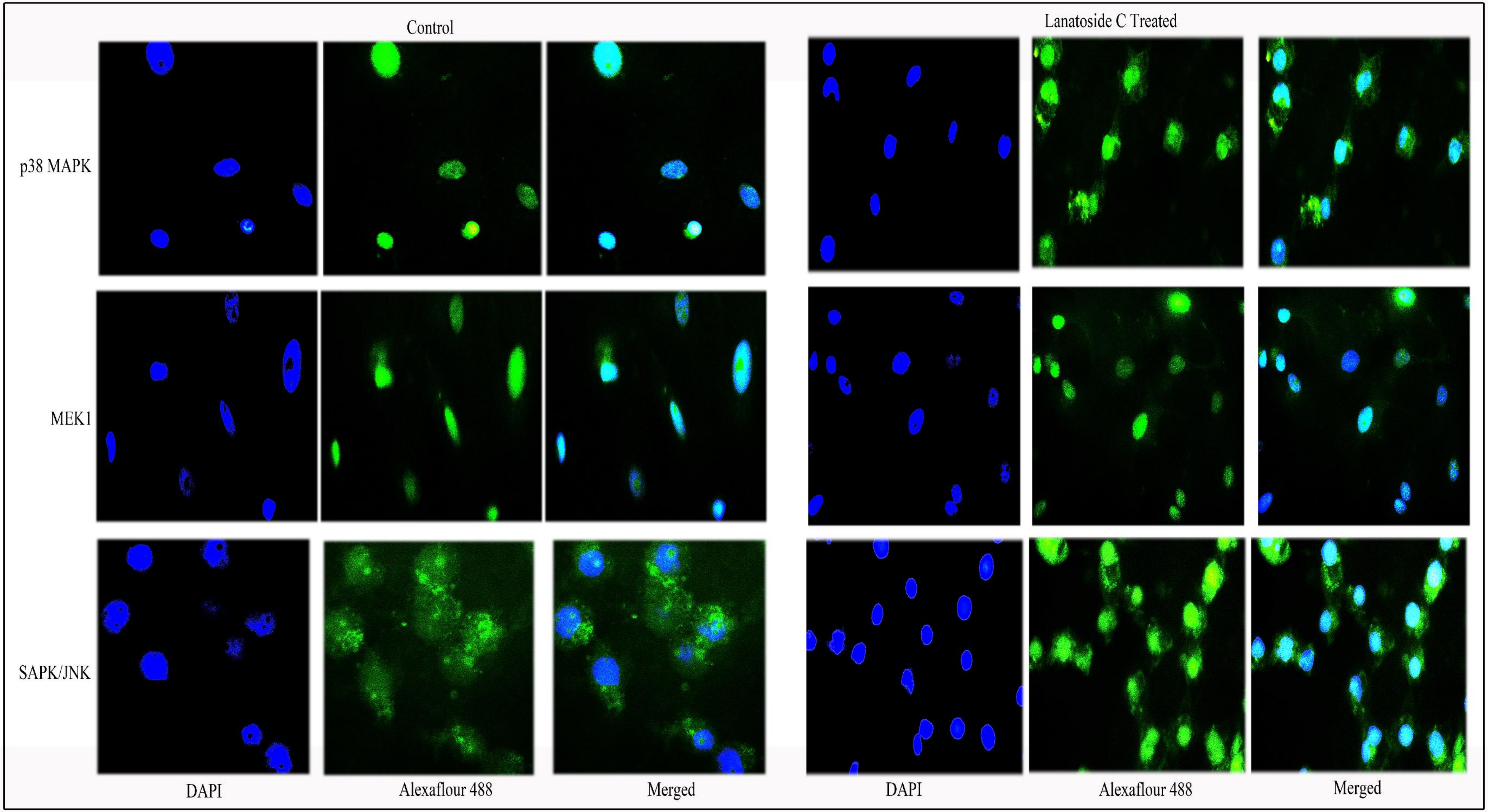

Supplementary Fig. 4C: Immunofluorescence imaging for the analysis of protein localisations of p38MAPK, MEK1and SAPK/JNK in Lanatoside C induced A549 cells.

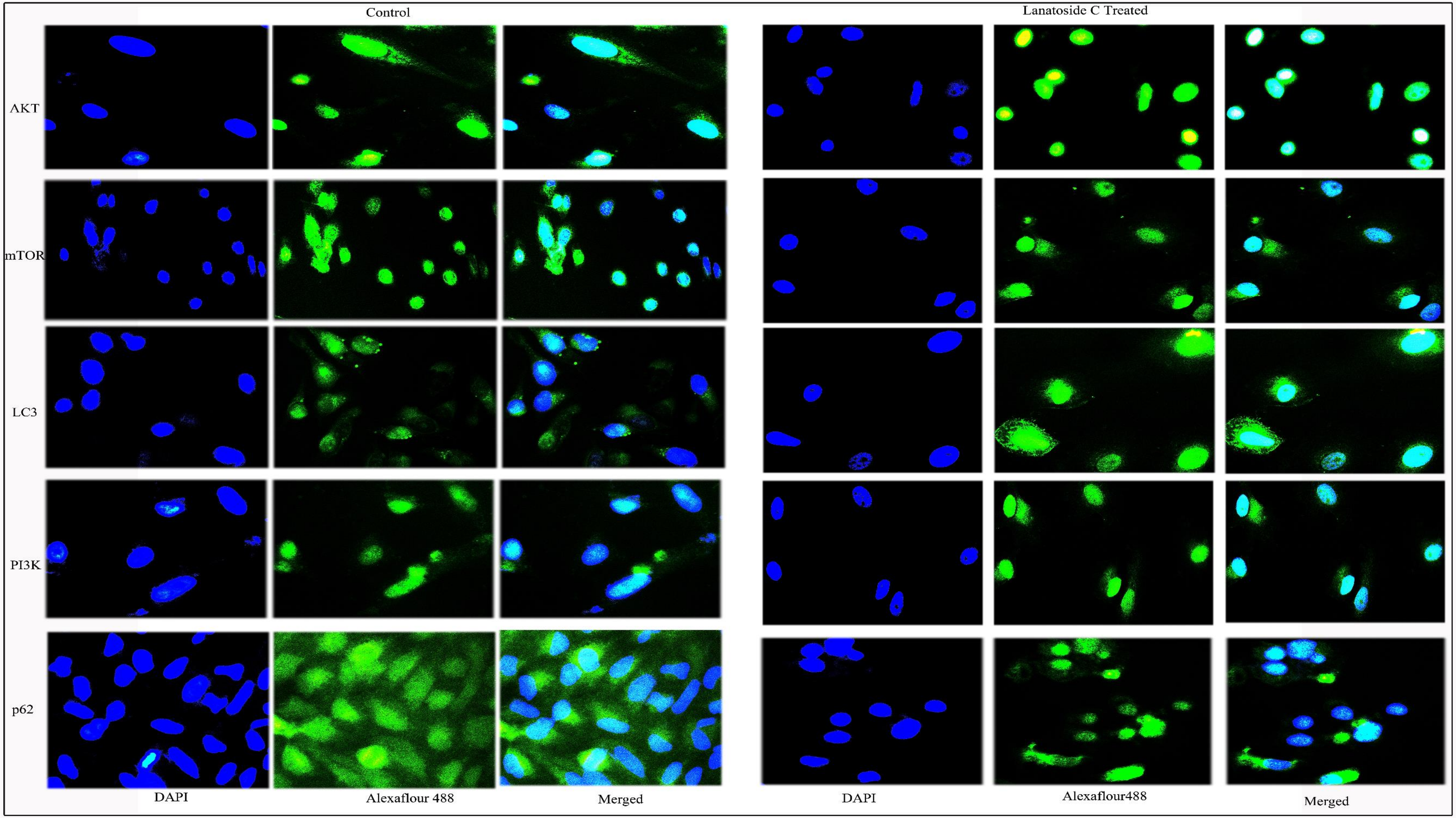

Supplementary Fig. 4D: Immunofluorescence imaging for the analysis of protein localisations in AKT, mTOR, LC3, PI3K and p62 in Lanatoside C induced A549 cells.

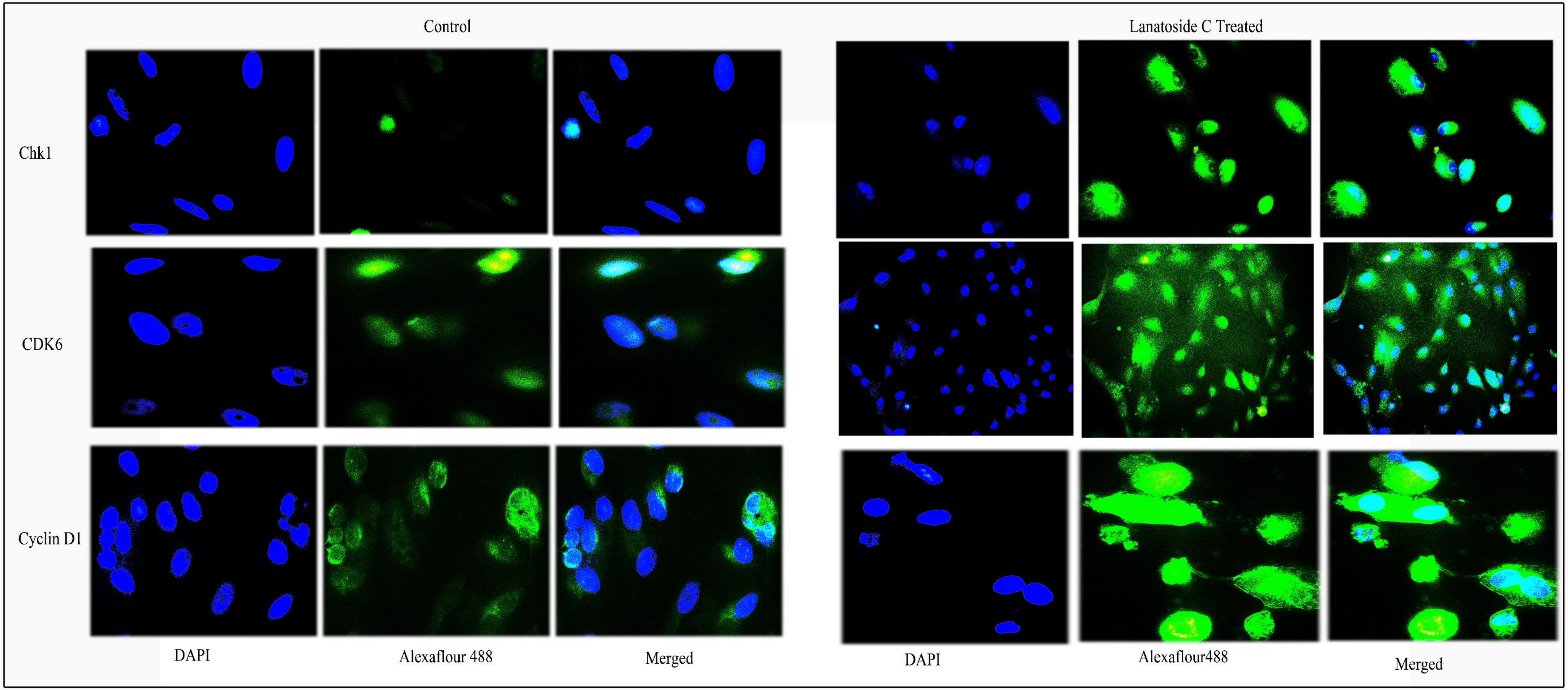

Supplementary Fig. 5A: Immunofluorescence imaging for the analysis of protein localisations of Chk1, CDK6 and Cyclin D1 in Lanatoside C induced A549 cells.

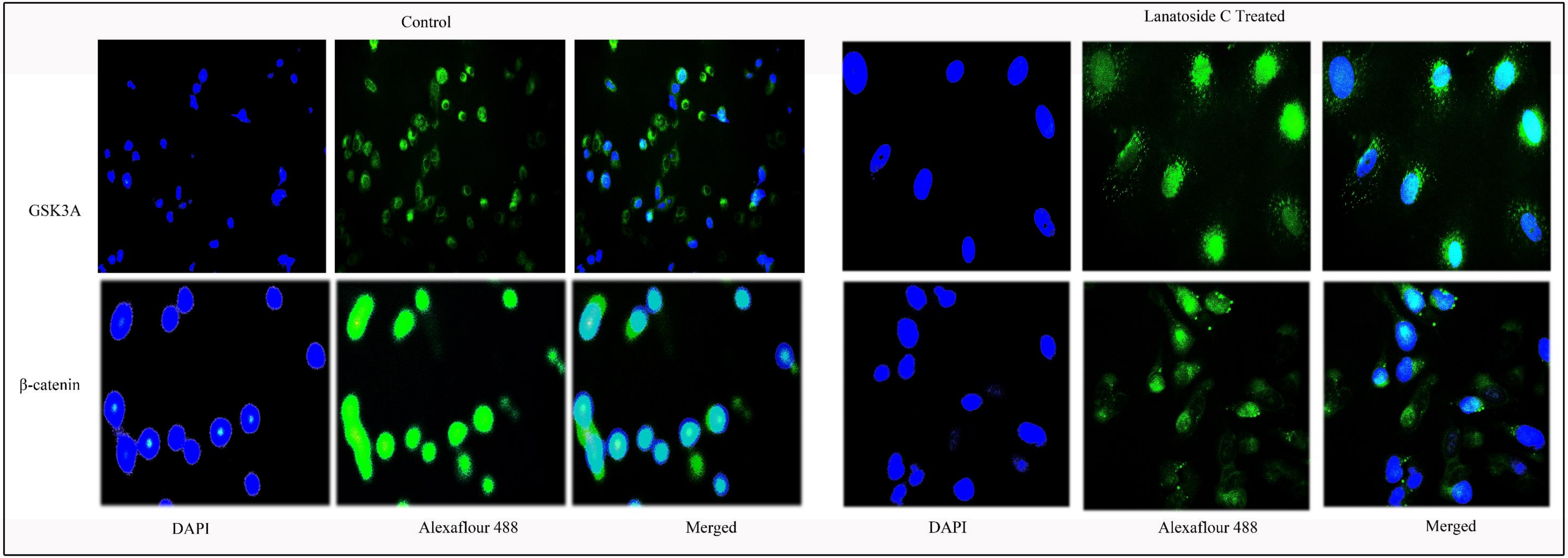

Supplementary Fig. 5B: Immunofluorescence imaging for the analysis of protein localisation of GSK3α and β-catenin in Lanatoside C induced HepG2 cells.

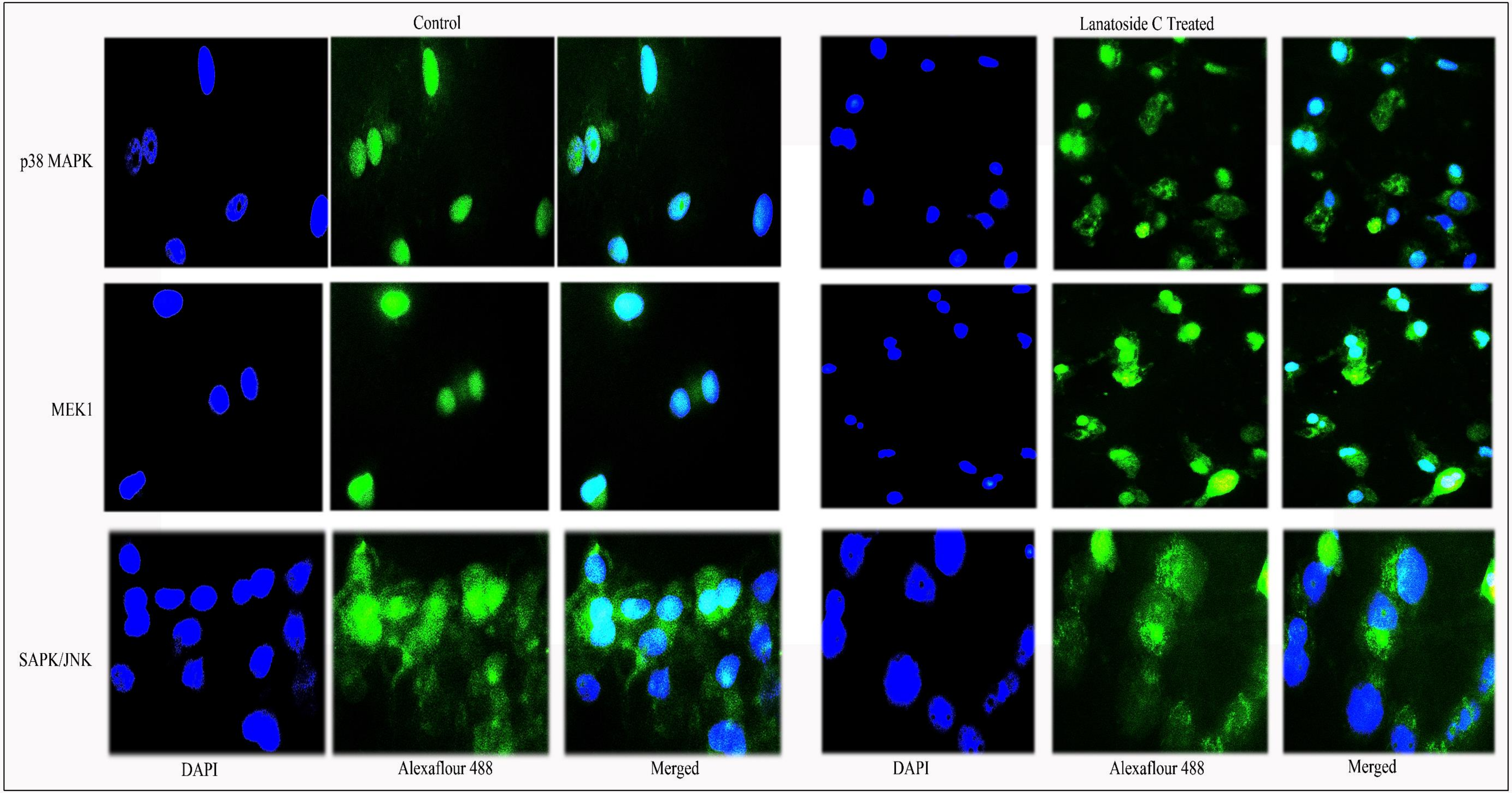

Supplementary Fig. 5C: Immunofluorescence imaging for the analysis of protein localisations of p38MAPK, MEK1and SAPK/JNK in Lanatoside C induced HepG2 cells.

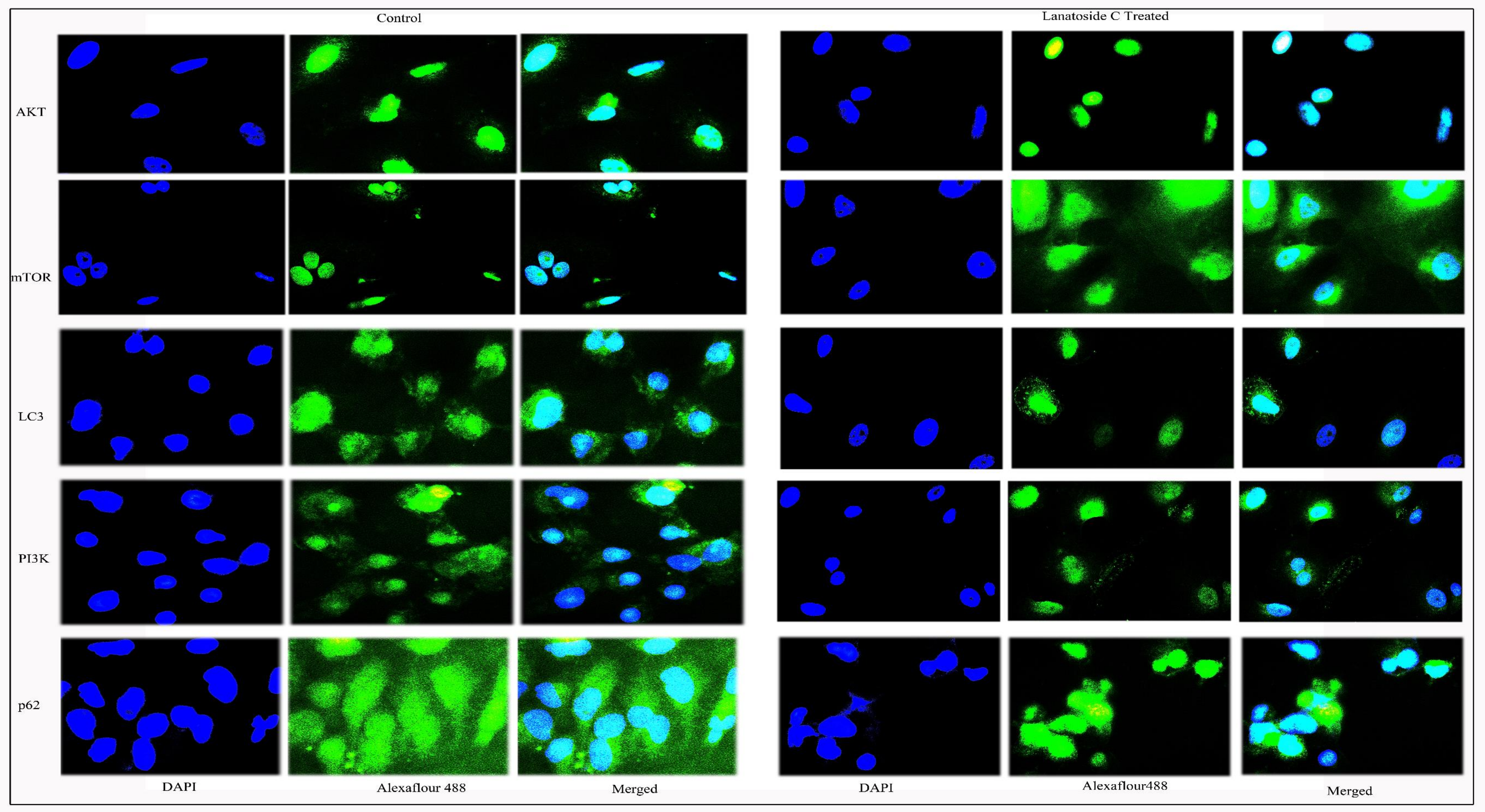

Supplementary Fig. 5D: Immunofluorescence imaging for the analysis of protein localisations in AKT, mTOR, LC3, PI3K and p62 in Lanatoside C induced HepG2 cells.

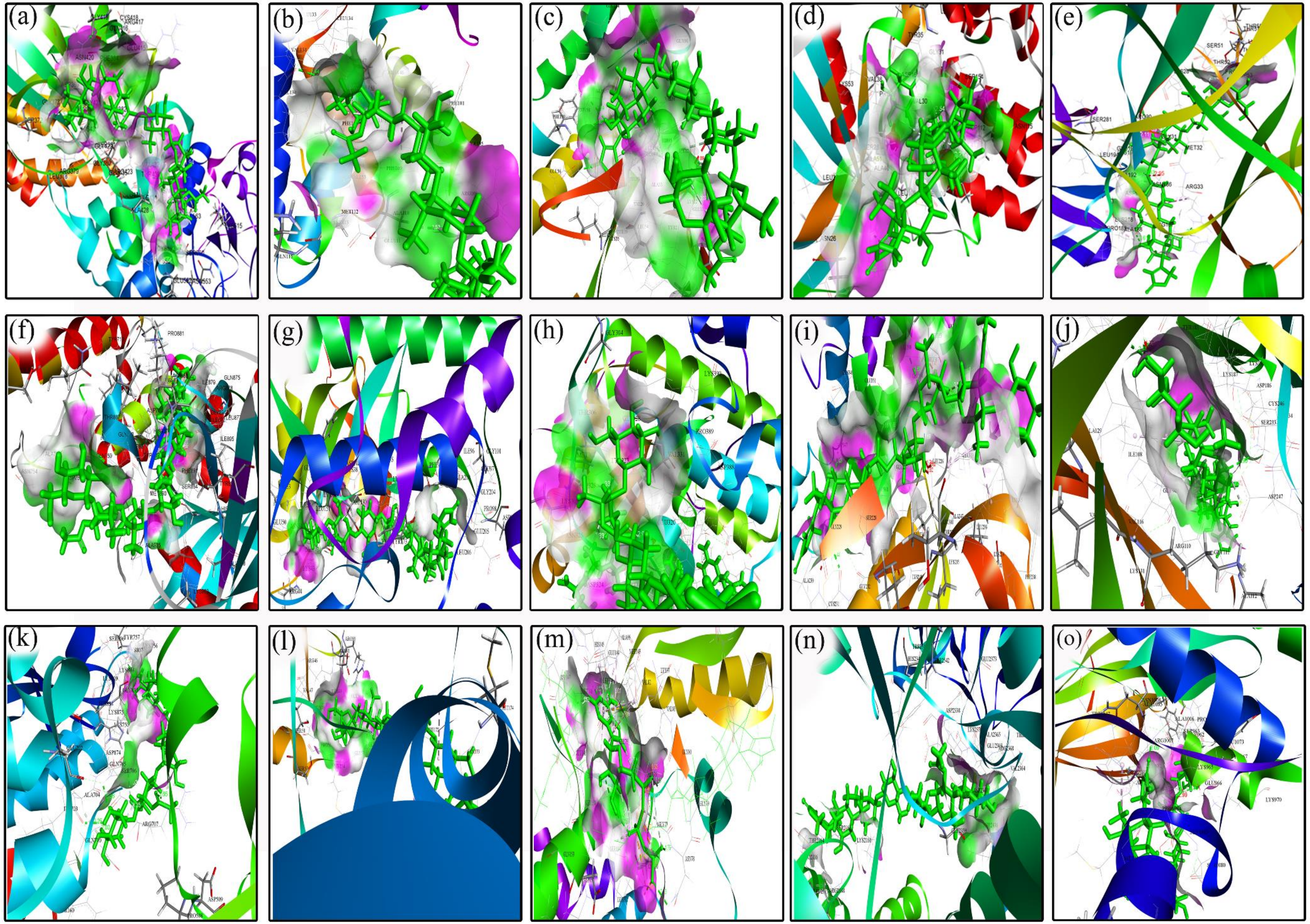

Supplementary Fig 6: Docking complexes of target protein and ligand (a) STAT3, (b) Bcl-2, (c) Cyclin D1, (d) p38, (e) NF-kB, (f)PARP, (g) Chk1, (h) AKT, (i) Chk2, (j) JNK, (k) PI3K, (l) CDK6, (m) MEK1, (n) mTOR, and (o) JAK.
